# PEGylated IL2 and tumor-targeted radiotherapy augment CD8^+^ T cell-mediated anti-tumor response to anti-PD-1 in head and neck squamous cell carcinoma

**DOI:** 10.7150/thno.135255

**Published:** 2026-08-12

**Authors:** Won Jong Jin, Hari Menon, Amber Bates, Lauren Zebertavage, Yujuan Wang, Paul A. Clark, Alexander A. Pieper, Caroline P. Kerr, Tracy J. Berg, Jens C Eickhoff, Sarah Seiter, A Mario Q. Marcondes, Mary A. Tagliaferri, Willem W. Overwijk, Tabassum A. Kennedy, Paul M. Sondel, Paul M. Harari, Justine Y. Bruce, Adam R. Burr, Reinier Hernandez, Irene M Ong, Jamey Weichert, Zachary S. Morris

**Affiliations:** 1 University of Wisconsin Madison, Department of Human Oncology, Madison, WI, USA.; 2 University of Wisconsin Madison, Department of Radiology, Madison, WI, USA.; 3 University of Wisconsin Madison, Department of Biostatistics and Medical Informatics, Madison, WI, USA.; 4 Nektar Therapeutics, San Francisco, CA, USA.; 5 University of Wisconsin Madison, Department of Pediatrics, Madison, WI, USA.; 6 University of Wisconsin Madison, Department of Medicine University of Wisconsin School of Medicine and Public Health,; 7 University of Wisconsin Madison, Department of Medical Physics; Madison, WI, USA.

**Keywords:** PEGylated IL2, radiation, anti-PD-1, memory CD8+ T cell, PD-L1+monocyte

## Abstract

Combination of immune checkpoint inhibitors (ICI) with either radiotherapy or PEGylated interleukin 2 (pegIL2) has produced limited or no benefits over ICI alone in clinical trials. A triple combination of radiotherapy, pegIL2, and ICI may overcome the limitations of dual treatments. We report a phase II clinical trial evaluating tumor-targeted radiotherapy, pegIL2 (BEMPEG), and ICI (pembrolizumab) in patients with recurrent or metastatic head and neck squamous cell carcinoma (HNSCC), and combination pre-clinically of radiopharmaceutical therapy (RPT), pegIL2, and ICI in HNSCC.

**Methods:**

Patients received one cycle of pegIL2 (0.006 mg/kg) plus pembrolizumab (200 mg) followed by external beam radiotherapy (EBRT), then two cycles of pegIL2 plus pembrolizumab. Adverse events, objective response rate (ORR), progression-free survival (PFS), overall survival (OS), and immune response from peripheral blood mononuclear cells (PBMCs) and biopsies were evaluated. RPT (^90^Y-NM600) was evaluated in combination with pegIL2 and anti-PD-L1 in murine MOC2 and SCC7 HNSCC models.

**Results:**

We report ORR of 40%, PFS of 4.2 months, OS of 13.6 months, and increased effector:regulatory T-cell ratios and memory CD8^+^ T cells in 5 patients treated prior to trial discontinuation. In preclinical HNSCC models, combined RPT and pegIL2 augmented memory CD8^+^ T cells (IL2Rβ^+^). CD8^+^ T cell depletion led to loss of anti-tumor responses. PegIL2 restored RPT-driven lymphocyte deficiency earlier, increasing memory CD8^+^ T cells (IL2Rβ^+^) in the tumor, and enhanced anti-tumor response through MHC I-dependent TNFα expression. A deficiency of the stimulator of interferon genes (STING) abrogated tumor MHC I expression and antitumor responses. PD-L1-expressing monocytes antagonized CD8^+^ T cell memory effect in the tumor recurrence stage, and anti-PD-L1 therapy promoted ^90^Y-NM600 and pegIL2 efficacy.

**Conclusions:**

Preclinically, tumor-targeted RPT and pegIL2 augment response to ICI through CD8^+^ T cell memory acquisition and regulatory monocyte suppression. This study provides preclinical and clinical insight for HNSCC treatment.

## Introduction

Immunotherapies such as immune checkpoint inhibitors (ICI) are effective in multiple cancer types. While they achieve durable responses in some patients, resistance remains amongst poorly immunogenic tumors, characterized by low levels of or exhausted T-cell infiltrates, low mutation burden, and few tumor-associated neoantigens 1. Radiation therapy (RT) is standardly used for treatment of head and neck squamous cell carcinoma (HNSCC) and can modulate tumor cell immune susceptibility as well as immune cell infiltration and activation in the tumor microenvironment (TME), thereby increasing responsiveness to ICI. Preclinically, we have observed this when combining tumor-targeted external beam radiation therapy (EBRT), which induced tumor antigen expression via STING-dependent type I interferon signaling, resulting in anti-tumoral T cell responses in a murine HNSCC model 2, 3. These combined treatments have been explored in multiple clinical trials 4, 5, but focal EBRT has failed to enhance response to anti-programmed death 1 (anti-PD-1) checkpoint blockade in patients with recurrent/metastatic (R/M) disease 6.

Inflaming the TME with a cytokine that increases expansion of T cells and other immune cells has been pursued with interleukin 2 (IL2). We have observed strong synergy between localized tumor EBRT and intratumoral IL2 injections in murine HNSCC 3. While IL2 has been investigated and used clinically for many years in cancer therapy, its use and effectiveness have been limited due to side effects, a short half-life, and its potential to stimulate regulatory T cells (Tregs) 3, 7-9. IL2 has a high preference for IL2 receptor α (IL2Rα/CD25), a stimulator of Tregs, and only moderate preference for IL2 receptor β (IL2Rβ/CD122), which stimulates expansion of T effector cells (Teffs) and NK cells 10. Bempegaldesleukin, a PEGylated IL2 (pegIL2), is an IL2Rβ-preferential agonist consisting of recombinant human IL2 conjugated to six releasable molecules of polyethylene glycol capable of binding to both mouse and human IL2Rβ 10, 11. Thus, the bempegaldesleukin formulation of pegIL2 provides preferential expansion of Teffs over Tregs while overcoming drawbacks of IL2 therapy by providing a longer half-life and reduced risk of dose-related toxicity 12. The phase I PIVOT-02 trial in advanced solid tumors combining pegIL2 and anti-PD-1 therapy demonstrated that this combination was well tolerated and increased Teffs 13. The phase III follow-up trial in patients with advanced melanoma did not show improved response compared to anti-PD-1 monotherapy 14. This suggests that cooperative therapeutic efficacy of pegIL2 and anti-PD-1 is limited. While anti-PD-1 therapy has shown substantial efficacy in melanoma, intrinsic and acquired resistance limits response in over half of patients 15, numbers that were replicated in the phase III trial both with the PD-1 monotherapy and the combination with pegIL2. While pegIL2 can contribute to increasing ratios of Teffs over Tregs and anti-PD-1 therapy can interrupt the PD-1/PD-L1 immune checkpoint, additional immunosuppressive pathways stimulated by tumors contribute to resistance through additional mechanisms beyond these interventions. Radiation, while potentially toxic to Teffs, can stimulate immune response by increasing immunogenicity in the TME 16. Radiation, however, can have drawbacks of stimulating upregulation of PD-L1 on tumor cells 17. A combination of radiation with pegIL2, which can preferentially stimulate Teffs over Tregs, and anti-PD-1, which can interfere with the PD-1/PD-L1 immune checkpoint, may therefore overcome these drawbacks and provide a deeper and more sustained immune response. Preclinical studies have demonstrated potential effective combination of pegIL2 with EBRT 18, 19, and preclinical studies combining pegIL2 with ICI and RT to a single tumor site provided promising results across solid tumor models 19. We therefore hypothesized that combination of RT, pegIL2, and anti-PD-1 can overcome the limited response achieved with combinations of either RT and anti-PD-1 or anti-PD-1 and pegIL2. This was tested in a small phase II clinical trial (NCT04936841), the results of which we report here. This trial evaluated safety, feasibility, and markers of immune response with combined pembrolizumab (anti-PD-1), pegIL2, and EBRT for patients with R/M HNSCC.

A critical limitation of EBRT is the inability to safely and feasibly target all tumor sites in settings of metastatic disease, necessitating a reliance on abscopal responses to achieve systemic anti-tumor response. Therefore, to evaluate a next generation approach to combining RT, pegIL2, and ICIs, in this study we examine the use of a novel radiopharmaceutical (RPT) delivering ^90^Yittrium (^90^Y, 2.67 days half-life) to all tumor sites following intravenous injection 20. As a tumor-targeting agent, NM600, an alkylphosphocholine–DOTA chelator, shows tumor-selective uptake and retention in solid tumors of nearly any type 21. ^90^Y-NM600 can elicit anti-tumor responses when administered in combination with ICIs 22. Similar to EBRT, ^90^Y-NM600 can stimulate type I interferon in murine melanoma and HNSCC models 23. Here, we tested the preclinical efficacy and safety of combining ^90^Y-NM600 with pegIL2 and ICIs in syngeneic murine models of HNSCC.

## Materials and Methods

### Clinical trial study design

A phase II study was conducted to assess the combination of palliative radiotherapy (24 Gy, in three daily 8 Gy fractions), pembrolizumab (anti-PD-1, 200 mg), and pegIL2 (bempegaldesleukin, 0.006 mg/kg^3^; Nektar Therapeutics). The University of Wisconsin Carbone Cancer Center (UWCCC) enrolled participants who had a histologically proven diagnosis of head and neck squamous cell carcinoma that was metastatic or recurrent disease, and were surgically incurable, and received written informed consent and HIPAA authorization. The targeted tumor was selected based on a size of at least 1 cm in the longest dimension, where the RT is deliverable.

### Blood specimens, inducible live single-cell multiplex secretome study, and polyfunctionality strength index

Whole blood was collected in Vacutainer CPT Mononuclear Cell Preparation Tubes (BD) at each cycle immediately before and 7 days after pegIL2 and pembrolizumab administration (d1 and d8). This timing was chosen to allow evaluation of pre- and post-treatment effects and comparison of these between cycle 1 (first treatment with pegIL2), cycle 2 (first treatment after radiation therapy), and cycle 3 (treatment with pegIL2 alone, but after having received radiation in cycle 2) The blood-containing tube was centrifuged at 1500 x g for 20 min at room temperature. Then, the PBMC layer was isolated and counted using auto cell counter Countess3 (Invitrogen). The cells were mixed with cryopreservation media containing 90% heat-inactivated fetal bovine serum (FBS, Life Technology) and 10% dimethyl sulfoxide (DMSO, Sigma-Aldrich) then stored in liquid nitrogen.

Single cells were prepared by thawing cryopreserved PBMCs and culturing these with complete media (RPMI 1640 (Corning), IL2 (10 ng/mL, eBioscience), 10% FBS (Life Technologies) and 100 U/mL penicillin/streptomycin, Life Technologies) at 5% CO_2_, 37 °C. After 24 h, CD8^+^ T cells were sorted using a microbead sorting kit (Miltenyi Biotec) and cultured on an anti-CD3 antibody-coated 96-well plate (10 µg/mL, eBioscience) with complete media containing anti-CD28 antibody (5 µg/mL, eBioscience). After 24 h, the cells were stained with Alexa Fluor 647 anti-CD8 antibody (PhenomeX) and loaded into an Adaptive Immune IsoCode chip (PhenomeX). Secretomes were detected during 16 h of incubation and analyzed by IsoPeak software. Polyfunctionality Strength Index (PSI) was calculated as the percentage of polyfunctional cells (two or more cytokine-secreting cells) multiplied by the sum of the median fluorescence intensities (MFIs) of the secreted cytokines by those cells.

### Biopsy immunostaining, multispectral imaging, and analysis

A research-related biopsy was performed by core needle, punch, or incisional techniques at the discretion of the surgeon/interventional radiologist before treatment initiation and at day 8 (± 5 days) of cycle 2. Histologic immunostaining was performed at the University of Wisconsin Translational Research in Pathology Core Facility. Biopsy tissues were formalin-fixed and paraffin-embedded (FFPE). FFPE tissue blocks were sectioned (5 μm) to slides. Automated immunohistochemistry was conducted on the Ventana Discovery Ultra BioMarker Platform (Ventana Medical Systems). Deparaffinization followed by heat-induced epitope retrieval utilizing cell conditioner 1 buffer (Ventana #950-224), an EDTA-based buffer with a pH of 8.4, heated for 64 min at 95 ℃ was performed by the instrument. Immunostaining was performed using recombinant anti-CD14 antibody (EFR3653, abcam), 1:100 anti-PD-L1 (28-8, abcam), 1:100 anti-IL2Rβ/CD122 (EPR24336-29, abcam), 1:500 anti-CD44 (EPR1013YM, abcam), 1:50 anti-FOXP3 (D2W8E, Cell Signaling), and pre-diluted anti-CD8 (SP239, Roche). All primary antibodies were diluted as indicated below in DaVinci Green antibody diluent (BioCare Medical #PD900H) and were incubated for 60 min at 37 ℃. Following this step, slices were rinsed with reaction buffer (Ventana #950-300), incubated with Discovery OmniMap anti-rabbit HRP (Ventana #760-4311) for 16 min at 37 ℃, rinsed with reaction buffer, then visualized using the Discovery ChromoMap DAB detection kit (Ventana #760-159). Additional steps for dual staining follow as indicated.

IL2Rβ and CD8 dual staining: Slides were incubated with IL2Rβ (Abcam #ab271040). After HRP visualization, a Discovery Inhibitor (Ventana #760-4840) denaturing agent was applied for the preset time before the addition of the second antibody, 100 l prediluted CD8 antibody (SP57 Roche diagnostic, # 790-4460). Following rinsing with reaction buffer (Ventana #950-300), slides were incubated with Discovery OmniMap anti-rabbit HRP (Ventana #760-4311) for 16 min at 37 ℃, rinsed, then visualized with the Discovery Purple HRP detection kit (Ventana # 760-229, 20 min).

PD-L1 and CD14 dual staining: Slides were incubated with a 1:100 dilution of the first antibody, PD-L1 (Abcam cat# ab205921). After HRP visualization, Discovery Inhibitor (Ventana #760-4840) denaturing agent was applied for the preset time before adding the second antibody, CD14 (Abcam cat# ab133335), for 32 min at 37 degrees. Following rinse (Ventana #950-300), slides were incubated with Discovery OmniMap anti-rabbit HRP (Ventana #760-4311) for 16 min at 37 ℃. After another rinse with reaction buffer, rinsed, then visualized with the Discovery Purple HRP detection kit (Ventana # 760-229, 12 min).

Upon completion of staining, slides were removed from the instrument, counterstained with Harris hematoxylin (diluted 1:5) for 45 s, rinsed with dH2O, then dehydrated by oven drying and subsequent dipping in xylene before applying the coverslip.

A Vectra multispectral slide scanner (Akoya) was used to scan 20x images from slides. Spectral library algorithms to unmix the image channels (DAB, Purple, and hematoxylin) were generated to determine quantitative protein analysis. Multiple 20x images per patient with at least 600 hematoxylin-positive cells were analyzed using inForm 1.4 software (Akoya).

### Cell lines

The murine HNSCC cell lines MOC2 and SCC7 were obtained from Dr. Ravindra Uppaluri (Brigham and Women’s Hospital and Dana-Farber Cancer Institute) and Dr. Stephen P. Schoenberger (La Jolla Institute). Lewis Lung Carcinoma (LLC) was obtained from American Type Culture Collection (ATCC, CRL-1642). The MOC2 cells were cultured in the media containing Dulbecco’s Modified Eagle Medium (DMEM; Corning) / Ham’s F12 (Corning) at a 2:1 mixture, 5% fetal bovine serum (FBS, Life Technologies), epidermal growth factor (5 ng/mL, Gibco), hydrocortisone (400 ng/mL, Sigma-Aldrich), insulin (5 μg/mL, Sigma-Aldrich), and 100 U/mL of penicillin/streptomycin (Life Technologies). SCC7 cells were cultured in RPMI 1640 (Corning) with 12.5% FBS (Life Technologies), 1 µg/mL hydrocortisone (Sigma-Aldrich), and 100 U/mL penicillin/streptomycin (Life Technologies). LLC cells were cultured in DMEM (Corning), 10% FBS (Life Technologies), 100 U/mL penicillin/streptomycin (Life Technologies). All tumor cells were incubated at 37 ℃ in 5% CO_2_. Cell authentication was conducted under ATCC guidelines through morphology monitoring, growth curve analysis, and mycoplasma testing within 6 months of use.

### Radionuclides and radiochemistry

The β^+^ emitter ^86^Yttrium (^86^Y, half-life = 14.7 h) was produced by separation from the ^86^SrCO3 as previously described 55. Briefly, ^86^Y was produced via proton beam (14.1 MeV) to enriched ^86^SrCO3 solid targets. Following RT, the targets were incubated at room temperature to decay for 4h, then dissolved it in 6 N of hydrochloric acid (HCl). The final ^86^Y was eluted by single column extraction resin with 0.1 M HCl. ^90^Y was purchased as ^90^YCl_3_, a clinical grade from Echert and Ziegler. The 2-(trimethylammonio)ethyl(18-(4-(2-(4,7,10-tris(carboxymethyl)-1,4,7,10-tetraazacyclododecan1-yl)acetamido)phenyl)octadecyl) phosphate (NM600) was obtained by Archeus Technologies (Madison, WI). The ^86/90^Y radiolabeling with NM600 proceeded by mixing 10 to 100 μg per mCi of NM600 and 5 to 10 mCi of ^86/90^Y in 0.1 M NaOAc buffer (pH 5.5) for 30 min at 90°C on the shaker (500 rpm). The labeled compounds were purified by a reverse-phase Waters Oasis HLB Light cartridge (Milford) then formulated in PBS containing 0.4% v/v Tween 20 and 0.4% NaCl.

### Tumor-bearing mice generation and treatment

Mice were housed in accordance with the Guide for Care and Use of Laboratory Mice and treatments were performed under a protocol approved by the University of Wisconsin Institutional Animal Care and Use Committee (protocol# M005670). Orthotopic tumor-bearing mice with MOC2 or SCC7 were generated by right buccal injection of 1 x 10^6^ cells with or without 2 x 10^6^ tumor cell injection into the right flank subcutaneously during anesthetization by isoflurane. Single tumor-bearing mice with MOC2, SCC7, LLC, or MOC2-STING^hKO^ (STING heterozygotic knockout MOC2 cell, previously generated in 2 were generated by subcutaneous injection of 2 x 10^6^ cells into the right flank of 6-8 week old female mice (Taconic, C57BL/6 for MOC2 and LLC, C3H/Hen for SCC7; syngeneic mice models). When the desired tumor size was reached (30-100 mm^3^), mice were randomly separated and re-housed in the cage (5 mice/cage). After labeling the individual mice, each treatment was given with individual number recording. During the blinded treatment record, mice were monitored and tumor growth and survival rate measured. Bilateral tumor-bearing models were generated using MOC2 tumor cells by subcutaneous injection into the right and left flank (2 x 10^6^ cells) and randomized when mean tumor volume reached 70-120 mm^3^.

The initial treatment day was defined as “day 1” for all *in vivo* data. ^90^Y-NM600 was injected via tail vein on day 1. PegIL2 (16 µg; bempegaldesleukin, Nektar) was injected intravenously, and anti-PD-L1 antibody (200 µg, BioXcell) was injected intraperitoneally on the indicated days. Tumor sizes were determined using digital calipers and calculated as (width^2^ x length)/2. After treatment initiation (day 1), tumor growth was measured at least twice per week until individual mice were euthanized upon observation of tumor size exceeding 20 mm diameter, hunched posture, or veterinary recommended by an independent animal health monitor for morbidity or moribund behavior.

### Immune cell depletion

Isotype control IgG, anti-CD8 antibody (clone# 2.43, 300 μg, BioXcell), anti-CD4 antibody (clone# GK1.5, 300 μg, BioXcell), or anti-NK1.1 antibody (clone# PK136, 50 μg, BioXcell) was injected intraperitoneally in tumor-bearing mice on days 5, 9, and 14. For monocyte depletion, 500 μg of isotype control IgG or anti-CSF1R antibody was injected intraperitoneally on day 15; thereafter, 200 μg of the same antibodies was injected every other day from day 17 to 25. Depletion efficacy was evaluated by flow cytometry in the tumor (CD8^+^ T cells) and in blood (CD4^+^ T cells, NK cells, and monocytes). The anti-TNFα antibody (XT3.11) for *in vivo* use was obtained from BioXcell.

### Mouse immunization test

Host immune memory acquisition was determined by tumors re-engrafted on the opposite flank on day 90 (left flank, 5 x 10^5^ cells) from the initial treatment with the same tumor cell line they had been cured of. Naïve mice were used as control and engrafted with the same tumor cells at the same time. Two weeks post-engraftment, the number of mice in which tumors were grown was recorded. Systemic effector memory CD8 T cells were assessed by intravenous injection of tumors (MOC2; 5 × 105 cells) in mice rendered tumor-free by ^90^Y-NM600 + pegIL2 or control tumor-naïve mice. 72 h after tumor cell administration, blood was collected via axillary bleed, incubated with RBC Lysis Buffer (BioLegend) for 10 min at room temperature, then filtered using a 70 μm strainer (Falcon). The cells were centrifuged at 300 x g for 10 min at 25 °C, and the single-cell suspensions were analyzed for effector memory T cells (CD44^+^CD62L^-^) among total live CD8^+^ T cells.

### Mouse imaging and biodistribution

Mice bearing MOC2 and SCC7 were intravenously injected ^86^Y-NM600 (9.25 MBq) as previously described 22, anesthetized with 2% isoflurane, and serial CT (80 KV peak, 1000 mA-s, 220 angles) with 80 million coincidence events static PET scan (350-650 keV) was obtained by Inveon microPET/microCT scanner (Siemens Medical Solutions, Knoxville, TN). CT images were used to generate a density map of dosimetry estimation, and PET scans were reconstructed by a 3D-ordered subset expectation maximization algorithm. To determine ^86^Y-NM600 uptake, mice were euthanized, and tumors and normal tissues weighed and quantified by Wizard 2 γ-counter (PerkinElmer) to calculate the percent injected activity per gram of tissue (%IA/g; mean ± SD).

### Tumor multiplex protein profiling

Tumors were weighed, then placed in a tube with 5 µl/mg of Cell Lysis Buffer (Cell Signaling Technology) containing ceramic beads (Fisher Brand), PMSF (Cell Signaling Technology), and HaltTM Phosphatase Inhibitor Cocktail (Thermo Fisher Scientific). The mixture was homogenized twice by a Bead Ruptor Elite (OMNI) for 30 sec. The homogenized mixture was subjected to the multiplex immunoassay (MILLIPLEX MAP Mouse Cytokine/Chemokine Magnetic Bead Panel, MilliporeSigma) to quantify concentrations of 32 cytokines/chemokines. After the multiplex proteins were measured on the MAGPIX system (MilliporeSigma), each protein concentration was interpolated from curves constructed by protein standards and individual protein MFI reads (MILLIPREX Analyst, MilliporeSigma).

### Immune cell infiltrations and tumor MHC I analysis

Tumor tissues were dissociated using a Miltenyi gentle-MACS Octo Dissociator for 30 min in the RPMI 1640 (Corning) media containing Dnase (500 µg, Sigma-Aldrich) and collagenase (5 mg, Sigma-Aldrich), then incubated with red blood cell lysis buffer (Sigma-Aldrich) for 10 min at room temperature. The single cells were spun down by centrifugation (930 x g, 5 min, room temperature), and the supernatant was removed before proceeding with cell staining. Prior to staining, cells were treated with CD16/32 antibody (BioLegend) for 30 min to prevent non-specific binding. The cells were stained initially with Ghost Red Dye 780 (Tonbo Biosciences) according to the manufacturer’s instruction. Cell surface antigens were labeled using fluorescence-conjugated antibodies at 4°C for 30 min. Internal antigens were labeled with BD Cytofix/Cytoperm according to the manufacturer’s instructions. Cell subsets was determined on the Attune Flow Cytometer (ThermoFisher) after bead compensation (UltraComp eBeadsTM, Invitrogen) and calibration (Ultra Rainbow bead, Spherotech) using fluorescence minus one (FMO) gating methodology, then analyzed using FlowJo Software (BD). The following flow cytometry antibodies were obtained from BioLegend: PE/Cyanine7 anti-CD45 (clone# 13/2.3), FITC anti-CD3 (clone# 17A2), BV510 anti-CD4 (clone# RM4-5), PerCP/Cyanine5.5 anti-CD8a (clone# 53-6.7), BV605 anti-NK1.1 (clone# PK136), BV421 anti-CD279 (PD-1, clone# RAMPI-30), APC anti-CD279 (PD-1, clone# RAMPI-30), BV711 anti-CD44 (clone# IM7), BV605 anti-CD44 (clone# IM7), BV711 anti-TNFα (clone# MP6-XT22), PE anti-TNFα (clone# MP6-XT22), PE/Dazzle 594 anti-CD274 (PD-L1, clone# B7-H1), BV605 anti-Ki-67 (16A8), PE anti-IL2Rβ/CD122 (TM-β1), PE-dazzle anti-IL2Rβ/CD122 (TM-β1), BV711 anti-CD25 (PC61), BV711 anti-CD11b (M1/70), BV605 anti-Ly6C (AL-21), AF700 anti-Ly6G (1A8), BV421 anti-CCR2 (SA203G1), APC anti-IFNγ (XMG1.2), BV711-CD44 (IM7), BV510-CD62L (DREG-56), and anti-H2 (MHC I) antibody (M1/42).

### Hematological toxicity evaluation

MOC2 tumor-bearing mice were randomized into treatment groups of vehicle control, ^90^Y-NM600, pegIL2, and ^90^Y-NM600 + pegIL2. During treatment, mouse weight was tracked and blood samples were collected via axillary bleed (40~80 μl) on the indicated day. Complete blood count (CBC) was performed using the VETSCAN HM5 hematology analyzer (Abaxis).

### *Ex vivo* immunity test

Spleens were collected from mice and mechanically disaggregated using a syringe plunger in the presence of RPMI 1640 (4 ml, Corning), filtered through a 70 μm strainer (Falcon), then centrifuged (300 x g, 10 min, 25 ͦ C). The cell pellet was resuspended in RBC Lysis Buffer (BioLegend) for 10 min at room temperature. After RBC lysis, CD8 T cells were sorted using the MACS cell separation system (Miltenyi Biotec) according to the manufacturer’s instructions.

Blood was collected from tumor-bearing mice via axillary bleed, incubated with RBC Lysis Buffer (BioLegend) for 10 min at room temperature then filtered using a 70 μm strainer (Falcon), and centrifuged at 300 x g, 10 min, 25 ^o^C. The single cell suspensions were subjected to myeloid cell or monocyte isolation using the MACS separation system (Miltenyi Biotec).

To test memory effects, the sorted CD8^+^ T cells (2 x 10^5^) were cultured alone or co-cultured with pre-plated MOC2 or SCC7 (2 x 10^4^, respectively), incubated in complete media (RPMI 1640 containing 10% heat-inactivated FBS and 100 U/mL of penicillin/streptomycin). After 24h incubations, the cells were collected and analyzed by flow cytometry as described above.

To test monocyte-mediated CD8^+^ T-cell antagonism, CD8^+^ T cells (2 x 10^5^) were co-cultured with pre-plated MOC2 cells (2 x 10^4^). Immediately, sorted monocytes suspended in complete media containing IgG or αPD-L1 antibody (2 μg/mL) were added to the CD8^+^ T cell + MOC2 cell co-cultured plate. After 24h incubation, total cells were collected and stained for flow cytometry as described above.

### PegIL2 cross-reactivity assay

Healthy donor PBMCs (Stem Cell) or mouse splenocytes (1 x 10^6^, respectively) isolated from naïve C57BL/c mice as described above were incubated in complete media (RPMI 1640 containing 10% heat-inactivated FBS and 100 U/mL of penicillin/streptomycin) in the presence of pegIL2 (0, 100, or 100 ng/mL). After 72h incubations, the cells were collected and IL2Rα expression quantified by flow cytometry as described above.

### Tumor MHC I rescue experiments

Memory-enriched CD8^+^ T cells were sorted from ^90^Y-NM600 + pegIL2-treated mouse spleens. The cells (2 × 10^5^) were co-cultured with pre-plated MOC2-WT or -STING-hKO in the presence of recombinant mouse IFNβ (BioLegend) at indicated doses (0, 50, or 150 ng/mL). After 24h incubation, the cells were collected and analyzed by flow cytometry to detect MHC I and annexin V (AV) MFI in CD8-tumor cells.

### Gene expression analysis

Tumors from either MOC2-WT or MOC2-STING-hKO were collected on day 17 after treatment in mice, then placed in tubes containing 2.8 mm ceramic beads (Fisherbrand) with 1 ml of TRIzol reagent (Invitrogen). The tumors were homogenized using a Bead Mill Homogenizer (Bead Ruptor Elite, Omni International) for 30 s, twice. Total RNA was extracted from homogenized samples using the RNeasy Mini Kit (QIAGEN) according to the manufacturer’s instructions. The extracted RNA concentration was determined using NanoDrop 10000 (ThermoFisher) and subjected to complementary cDNA synthesis using the QuantiTect Reverse Transcription Kit (QIAGEN) according to the manufacturer’s instructions. Quantitative polymerase chain reaction (qPCR) was conducted using cDNA, PowerUp SYBR Green qPCR Master Mix (Applied Biosystems), and primers. Ct values were calculated using the ∆∆Ct method with HPRT as endogenous control. Primer sequences are listed in Table S1.

### IFNβ tumor-secretome and blood IL2 measurement

Tumor tissues were weighed then dissociated using the Tumor Dissociation Kit (Miltenyi Biotec) according to the manufacturer’s instructions. Dissociated tumors were filtered using a 70 μm strainer (Falcon) and centrifuged at 300 x g for 10 min. Supernatants were collected and IFNβ secretomes measured using a mouse IFNβ ELISA kit (BioLegend). For *in vitro* measurement, MOC2-WT or MOC2-STING hKO cells (2 x 10^5^) were cultured in 6-well plates. After 24h incubation, ^90^Y-NM600 was administered followed by 7 days of incubation. Then, the supernatants were collected and centrifugated at 2,800 x g, 10 min, 4 ^o^C. The upper layer was collected and IFNβ secretomes measured as described above.

Blood samples were collected via axillary bleed in the capillary blood collection tube (Separator Gel Additive, BD). The tubes were centrifuged at 2,800 x g for 10 min at room temperature. Separated serum was collected and used to quantify human IL2 using a human IL2 ELISA kit (BioLegend).

### Monocyte migration assay

Freshly collected blood from tumor-bearing mice was placed in Microtainer (BD) tubes and centrifuged at 1,300 x g for 10 min. The serum in the upper layer was collected and 50 μl was mixed with 450 μl of serum-free RPMI 1640 (Corning). The prepared serum mixture was placed in the lower chamber of a 24-well transwell plate (Costar). Freshly sorted myelocyte or monocytes (5 x 10^4^) sorted from another cohort of tumor-bearing mice were prepared in 300 μl of serum-free RMPI 1640 (Corning) and transferred into the upper chamber (5 μm-pore size, Costar). The plates were incubated for 5 h in a 37°C incubator. The number of cells that migrated to the lower chamber were determined using the Countess automated cell counter (Invitrogen).

### Statistical methods

Tumor growth was analyzed with a linear mixed effects model with animal-specific random effects and an autoregressive correlation structure to account for repeated measures over time. Tumor volume measures greater than 0 were log-transformed prior to conducting the analyses. In cases where the tumor volume was 0, a value of 0 + e (e = 0.001) was used before performing the log-transformation. The interaction effects between group and time (day) were evaluated and sliced contrasts were used to conduct comparisons between groups at various time points (days). Tukey's Honestly Significant Difference (HSD) method was utilized to control the type I error (< 0.05) when conducting multiple pairwise comparisons between groups. All reported P-values are two-sided, and p < 0.05 was used to define statistical significance with ∗ ≤ 0.05; ∗∗ ≤ 0.01; ∗∗∗ ≤ 0.001. Statistical analyses were conducted using SAS software (SAS Institute, Cary NC) version 9.4 or GraphPad Prism version 10. Survival probability was estimated and plotted using the Kaplan-Meier method and compared using the log-rank test. Observed differences among three or more groups were compared using ANOVA and Tukey’s method for multiple comparisons. Two-tailed Student’s t-test was used for a two-sample comparison. Linear regression analysis with two-tailed test was used for mRNA correlation analysis. Data indicate the mean value, and error bars indicate standard deviation (SD). All experiments containing at least three samples per group and were replicated to confirm reported observations. Data from the first or combined replicates are shown.

### Study approval

The phase II clinical trial UW20092 was approved by the Health Science Institutional Review Board (IRB) that serves the University of Wisconsin Hospital and Clinics (NCT04936841). Written informed consents were obtained from all participants. All mice were housed and managed in accordance with the Guide for Care and Use of Laboratory Mice. The animal studies were conducted under a protocol (#M005670) approved by the University of Wisconsin Institutional Animal Care and Use Committee 13.

## Results and Discussion

Five patients with surgically incurable R/M HNSCC were enrolled in a single-arm Phase II study between August 1, 2021, and February 1, 2022, at the University of Wisconsin Carbone Cancer Center (Table S2). The study was terminated early due to discontinued provision of pegIL2 by Nektar Therapeutics. Patients received one cycle of pegIL2 (0.006 mg/kg) plus pembrolizumab (200 mg) followed by palliative external beam radiotherapy (24 Gy in three daily 8 Gy doses targeting one or more tumor sites, with at least one site not radiated), then two cycles of pegIL2 plus pembrolizumab. Patients were assessed for adverse events (AEs) and completed the European Organization for the Research and Treatment of Cancer Quality of Life Questionnaire (EORTC-QLQ) (Table S3-S6). All patients reported at least one immune-related AE, with the majority reporting Grade 1 AEs. Grade 3 or 4 treatment-related AEs were reported in 20% (1/5) of patients reporting and no patients experienced grade 5 treatment-related toxicity. One patient experienced grade 3 hyperthyroidism and a grade 4 neutrophil count decrease. Two patients experienced lymphocyte count decrease and eosinophilia was noted in 4/5 patients, which are common IL2-driven AEs 24.

### Tumor radiotherapy augments polyfunctional and memory T cells response upon pegIL2 and pembrolizumab combination

**Figure 1:**
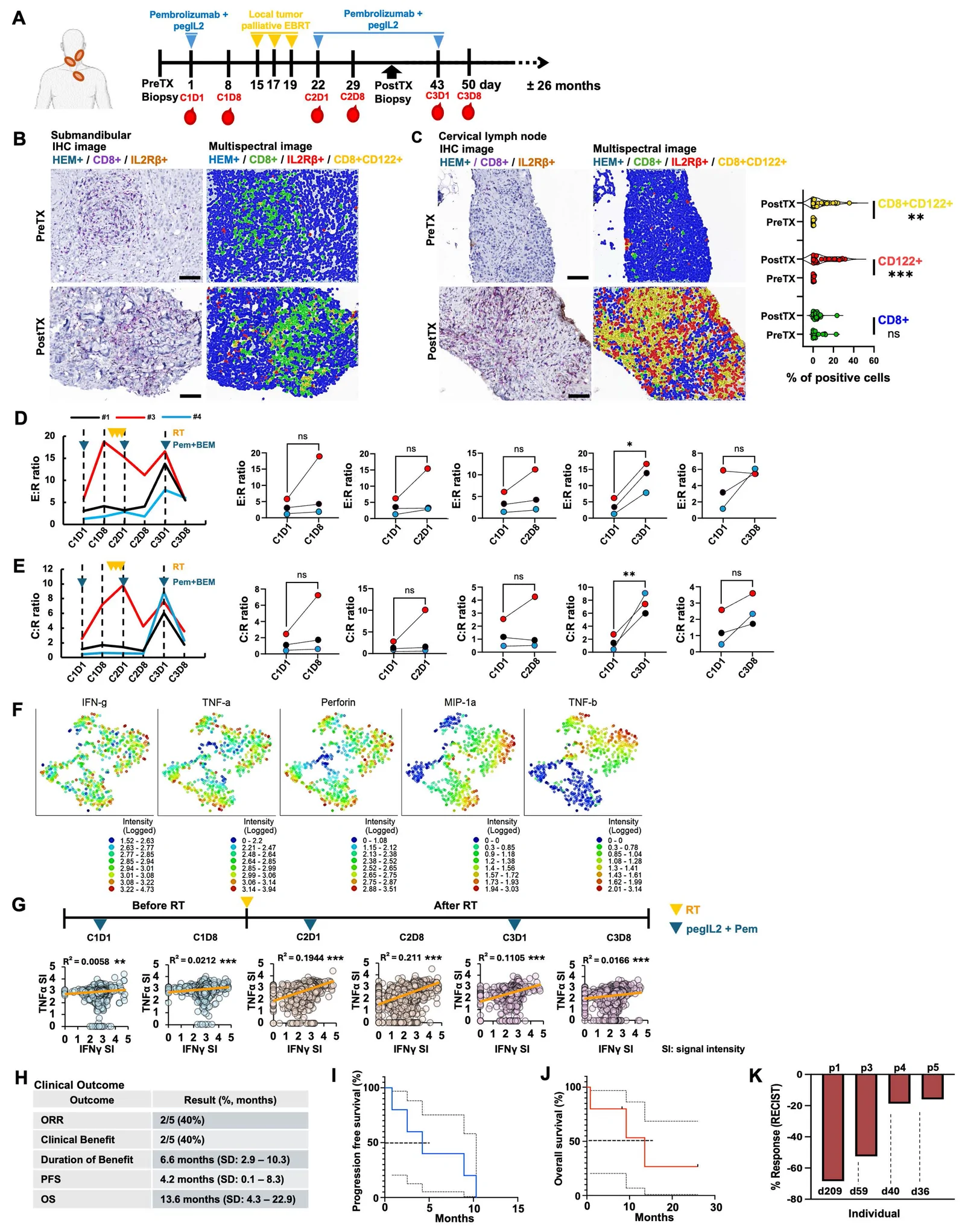
** Tumor radiotherapy augments polyfunctional and memory T cells response upon pegIL2 and pembrolizumab combination.** (**A**) Treatment and specimen collection scheme. (**B**) Multispectral immunohistochemistry analysis (CD8^+^, IL2Rβ^+^) in submandibular mass (left) and cervical lymph node (right). ∗∗ ≤ 0.01; ∗∗∗ ≤ 0.001 by two-sided T-test. **(C)** Combined quantification of cell types across all regions analyzed from 3 patients. HEM; hematoxylin, scale bar is 50 µm. **D-G**, Single-cell secretome analysis of CD8^+^ T cells isolated from PBMCs collected from cycles indicated in (A). (**D**) Effector:regulatory (E:R) and (**E**) chemoattractive:regulatory (C:R) ratios at indicated cycles. (**F**) Polyfunctional T cell t-SNE plots. **(G)** Protein correlation analysis. (**H**) Clinical outcome at six months after treatment initiation. ORR, overall response rate; PFS, progression-free survival; OS, overall survival. (**I**) Progression-free survival curve. (**J**) Overall survival curve. (**K**) Target lesion change (%) from individual patients at indicated day measured.

To evaluate treatment-driven CD8^+^ T cell responses, blood and tumor biopsies were obtained per protocol (Figure 1A). These were available for three subjects and were unavailable for one subject who died prior to initiating treatment (#2) and one who declined biopsy (#5). While the total number of infiltrating CD8^+^ T cells was unchanged, multispectral IHC analysis demonstrated increased memory CD8^+^ T cell infiltration (CD8^+^IL2Rβ^+^ and CD8^+^CD44^+^ cells) in PostTx compared to PreTx in tumor biopsies (Figure 1B-C and Figure S1A) 25. Given the increased memory CD8^+^ T cell tumor infiltration, we asked whether the treatment elicited a systemic immune response. CD8^+^ T cells from blood specimens across the treatment time course were subjected to single-cell proteomics to assess cytokine production. The expression levels of 32 cytokines were assessed in CD8+ T cells from each sample collection in patients #1, #3, and #4. These 32 cytokines were categorized as effector, chemoattractive, stimulatory, inflammatory, or regulatory, and their signal intensity was used to assess the predominant phenotype within each patient (Figure S1B). Single cells were further assayed for polyfunctionality by quantifying those exhibiting expression of multiple cytokines (Figure S1C). The polyfunctional strength index (PSI) measures release of multiple cytokines from individual CD8^+^ T cells and may reflect their potency and capacity to produce immune response 26. PSI of the CD8^+^ T cells in each category at each time point was quantified (Figure S1D) and the effector:regulatory (E:R; Figure 1D), chemoattractive:regulatory (C:R; Figure 1E), and stimulatory:regulatory (S:R; Figure S1E) ratios were analyzed. These ratios increased significantly from baseline (cycle 1, day 1; C1D1) in all patients at C3D1. Notably, this increase occurred after the combined pegIL2 + pembrolizumab treatment post-RT but was not observed at C1D8 after the pegIL2 + pembrolizumab treatment that occurred prior to RT.

Cytokine signal intensity from C3D1 CD8^+^ T cells was used to generate a t-SNE map, demonstrating distinct cytokine expression profiles amongst subsets of these CD8^+^ T cells (Figure 1F). Notably, some cytokines were almost universally enriched, such as IFN, TNF, and PERFORIN, while others were only elevated in limited subsets of CD8^+^ T cells. Interestingly, correlation between the expression of some cytokines was increased within single cells post-RT therapy. The correlation between the well-known tumor-antagonists TNFα and IFNγ 27 being expressed in a single cell increased post-RT compared to pre-RT, with the highest R^2^ value detected at C2D8, a significant increase compared to samples from the pre-treatment timepoint (C1D1) or after only pegIL2 + pembrolizumab (C1D8) (Figure 1G). The correlation between single cells expressing both IFNγ and PERFORIN was similarly elevated at the post-RT C2D8 time point and gradually declined again by C3D8 (Figure S1F). These data suggest that RT increases the effector:regulatory ratio of cytokines from CD8^+^ T cells.

Treatment responses were evaluated using standard-of-care follow-up imaging and were based on RECIST 1.1 criteria. Overall response criteria six months after treatment initiation indicated that two patients met the criteria for clinical benefit per trial and two patients had stable disease (SD) that progressed. The investigator-assessed objective response rate (ORR) was 40% with partial response (PR) in these subjects (Figure 1H). The median progression-free survival (PFS) was 4.2 months, and the median overall survival (OS) was 13.6 months (Figure 1I-J). Patient 2 (P2) died early after cycle 1 and was excluded from further analyses. The percentage change in the target lesion was recorded for the remaining four patients and all experienced lesion size reduction (Figure 1K).

While patient numbers in this study were small due to the discontinuation, limiting statistical analysis of responses, our observations suggest that the combination of EBRT, pegIL2, and pembrolizumab has the potential to promote polyfunctional effector T-cell development. Compared to early cycles of pembrolizumab + pegIL2 without RT, after addition of RT to the therapy regimen, changes emerged in CD8^+^ T cells that suggest that this therapy shows promise of a beneficial immune response that would benefit from further study. However, these benefits generally weakened by day 50 (C3D8). We hypothesized that this may result from the suppressive effects of non-radiated tumor sites on the adaptive anti-tumor immune response. We have previously observed such effects with EBRT-based in situ vaccination 28 and observed that this could be overcome by delivering low dose radiation to all tumor sites using RPT 29.

### ^90^Y-NM600 RPT targets HNSCC with early hematological toxicity in mice

**Figure 2:**
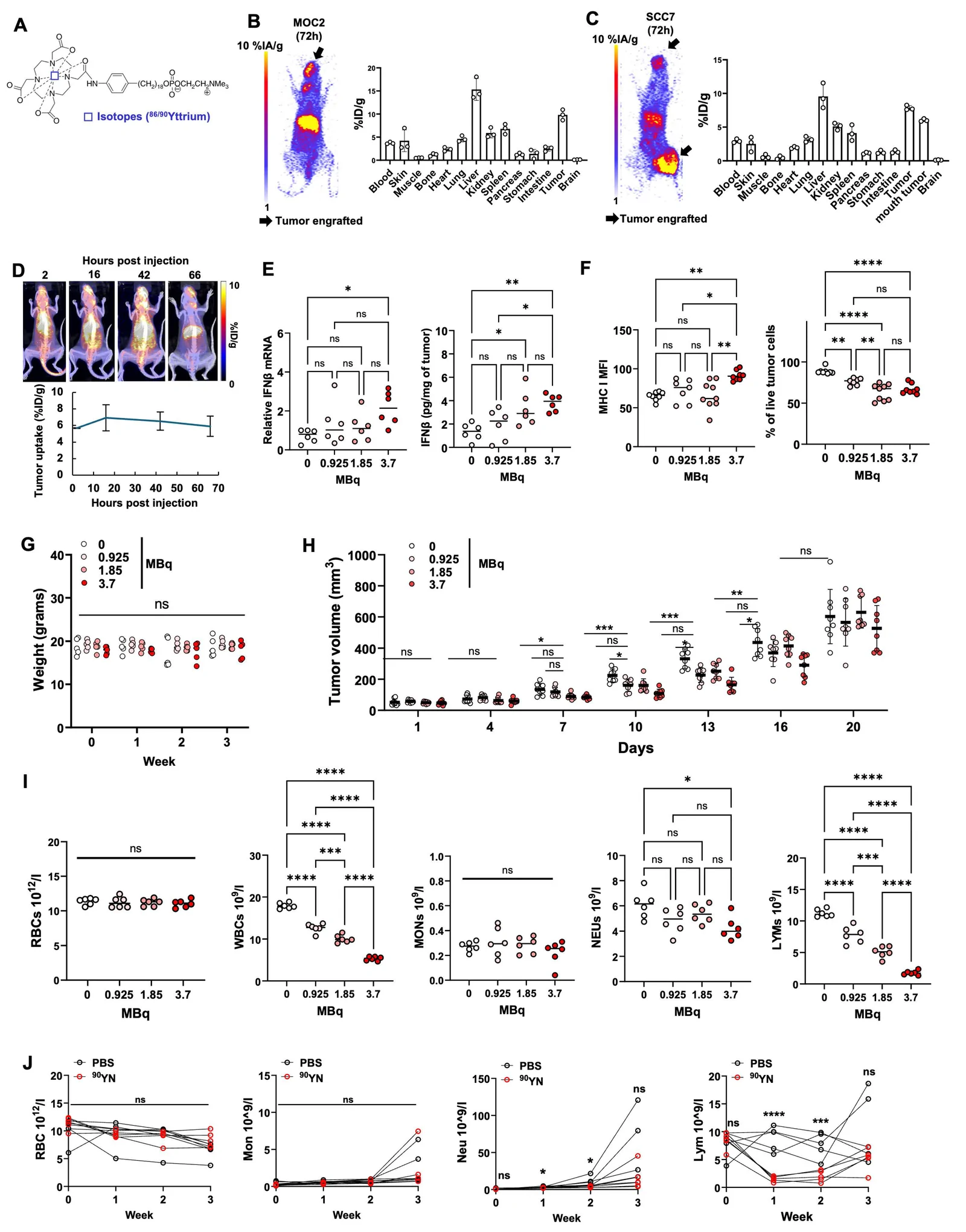
**^90^Y-NM600 RPT targets HNSCC with early hematological toxicity in mice.** (**A**) Schematic representation of NM600 and ^86/90^Yttrium radiolabeling. **B** and **C**, PET/CT imaging and biodistribution analysis of radioactivity uptake per gram of tissue (%IA/g) at 72 ho after intravenous injection of ^86^Y-NM600 in (**B**) MOC2 (cheek) or (**C**) SCC7 (cheek and right flank) (n = 3). Arrow bar indicates tumor location. (**D**) PET/CT imaging and percent tumor-uptake of injected dose per gram (%ID/g) over time. (n = 3/group) (**E**) Tumor IFNβ mRNA expression (left) and secreted IFNβ (right) analysis on day 7 post ^90^Y-NM600 treatment in mice with a MOC2 flank-tumor. (n = 6/group) (**F**) MHC I median fluorescence intensity (MFI) analysis in live^+^CD45^-^ cells (tumor) and % of live tumor cell analysis. (n = 7-9/group) (**G**) Whole body weight over time. (n = 5/group) (**H**) Tumor volume over time. (n = 8/group) **I** and **J**, Complete blood count (CBC) of red blood cells (RBCs), white blood cells (WBCs), monocytes (MONs), neutrophils (NEUs), and lymphocytes (LYMs) on (**I**) day 7 at the indicated dose (n = 6/group), or (**J**) over time after 3.7 megabecquerel (MBq) treatment. (n = 5/group) *P* values: ∗ ≤ 0.05; ∗∗ ≤ 0.01; ∗∗∗ ≤ 0.001 by (E-I) one-way ANOVA with Tukey’s correction for multiple comparisons or (J) unpaired T test. Error bars: Standard deviation.

We hypothesized that RPT therapy, which can target all sites of disease in metastatic settings, could produce effective T-cell activation, potentially yielding therapeutic benefit when combined with pegIL2 and ICI. We therefore performed pre-clinical testing of an RPT in combination with pegIL2 to determine whether combination of these therapies has the potential for therapeutic efficacy. The radionuclide yttrium (Y) can be used for diagnostic imaging (^86^Y, half-life 14.74 h) or therapeutic radiation delivery (^90^Y, half-life 2.67 days), which provides prolonged radiation delivery 30. Either Y isotope can be conjugated to alkylphosphocholine (NM600; Figure 2A) for tumor targeting 31. We investigated the feasibility and efficacy of ^90^Y-NM600 in combination with pegIL2 and ICI in preclinical HNSCC models. Positron emission tomography/computed tomography (PET/CT) imaging showed tumor-selective uptake and retention of intravenously administered ^86^Y-NM600 in orthotopic MOC2 and SCC7 HNSCC-bearing mice (Figure 2B-C). Biodistribution analyses of radioactivity revealed higher tumor uptake compared to immune cell-harboring tissues like blood, bone marrow, and spleen (Figure 2B-C). Although liver uptake of ^86^Y-NM600 was detected, the 3.7 MBq ^90^Y-NM600 used in these studies did not result in histological evidence of liver toxicity at day 25 post-administration (Figure S2A). Imaging-based tumor-uptake analysis showed prolonged retention of ^86^Y-NM600 in tumor until at least 66 h post injection (Figure 2D).

Given that RT induces tumor immune susceptibility through interferon β (IFNβ) and MHC I expression 32, 33, we asked what dose of therapeutic ^90^Y-NM600 would be required to induce IFNβ in tumors. Dose-escalated ^90^Y-NM600 administration increased IFNβ mRNA and protein secretion at the 3.7 MBq dose on day 7 post-treatment (Figure 2E). 3.7 MBq ^90^Y-NM600 increased tumor cell MHC I expression while reducing live tumor cells in both MOC2-bearing mice (Figure 2F and S2B [gating strategy]) and SCC7-bearing mice (Figure S2C). Body weight was unchanged over 3 weeks, suggesting tolerance of potential treatment-related toxicities of ^90^Y-NM600 in MOC2-bearing mice (Figure 2G). 3.7 MBq reduced tumor volume from day 7 to day 16, but no difference was observed at day 20 (Figure 2H). The red blood cell (RBC) count did not change on day 7, whereas the white blood cell (WBC) count significantly decreased in a dose-dependent manner with ^90^Y-NM600 (Figure 2I). Amongst WBC, monocytes were unchanged while neutrophils and lymphocytes decreased with 3.7 MBq. Lymphocytes demonstrated the greatest decrease and strongest dose response (Figure 2I). These data suggest that despite the gross tolerability of ^90^Y-NM600, hematological toxicities are present. Given that 3.7 MBq induced MHC I but also reduced white blood cell counts, we assessed the duration of this toxicity, finding that reduced neutrophil and lymphocyte counts persisted until week 2 but recovered in week 3 post-^90^Y-NM600 (3.7 MBq) administration (Figure 2J). These data suggest that ^90^Y-NM600 targets tumors and promotes greater immune susceptibility of the TME, but that hematologic toxicity could threaten its effectiveness in inhibiting tumor growth at higher dose levels.

### ^90^Y-NM600 and pegIL2 promote anti-tumor response against HNSCC murine models

**Figure 3:**
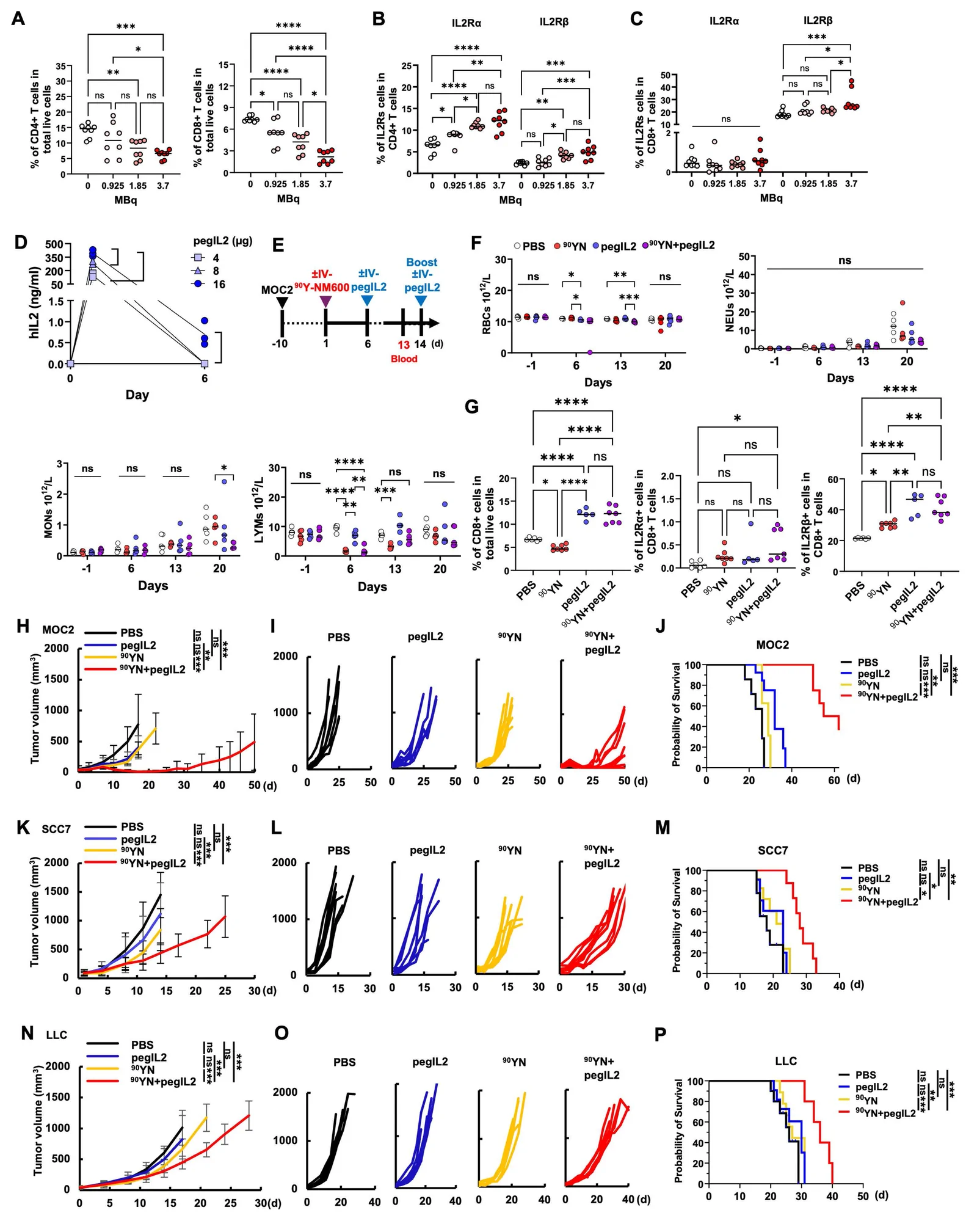
**^90^Y-NM600 and pegIL2 promote anti-tumor response.** Tumor-infiltrated (**A**) CD4^+^ T cells (left) and CD8^+^ T cells (right), (**B**) IL2Rα/β-expressing CD4^+^ T cells (left) and (**C**) CD8^+^ T cells (right) on day 7 post ^90^Y-NM600 (^90^YN) treatment in MOC2-bearing mice. (n = 8/group) (**D**) Quantification of human IL2 (hIL2) in serum after pegIL2 administration in MOC2-bearing mice. (n = 3/group) (**E**) Treatment scheme. (**F**) Complete blood count (CBC) of red blood cells (RBCs), neutrophils (NEUs), monocytes (MONs), and lymphocytes (LYMs) over time. (n = 5/group) (**G**) Blood CD8^+^ T cell analysis on day 13 post treatment. (n = 8-9/group) **H**-**P**, Mean tumor volume curves, individual tumor growth, and survival curves from (**H**-**J**) MOC2 (n = 7-8), (**K**-**L**) SCC7 (n = 7-8), and (**N**-**P**) LLC (n = 6). *P* values: ∗ ≤ 0.05; ∗∗ ≤ 0.01; ∗∗∗ ≤ 0.001 by (A-G) one-way ANOVA with Tukey’s correction for multiple comparisons; (K, N) linear mixed effects model with animal-specific random effects and an autoregressive correlation structure to account for repeated measures over time; (M, P) Log-rank test. Error bars: Standard deviation.

Given that ^90^Y-NM600 induced lymphocyte toxicity, we further explored CD4^+^ and CD8^+^ T cell lymphocyte populations. We observed a dose-dependent reduction in both live CD4^+^ and CD8^+^ T cells in blood with the ^90^Y-NM600 treatment (Figure 3A). This finding suggests co-administration of a drug that promotes T cell survival may prolong immune-related anti-tumor efficacy of ^90^Y-NM600. IL2 signaling can promote T cell survival, and pegIL2 prolongs IL2 effects in lymphocytes 10, 34. We hypothesized that pegIL2 would provide durable lymphocyte viability with ^90^Y-NM600. Interestingly, ^90^Y-NM600 increased expression of the IL2 receptors IL2R-α and -β in circulating CD4^+^ and CD8^+^ T cells, suggesting increased responsiveness to IL2 (Figure 3B-C, Figure S3A for flow cytometer gating strategy). PegIL2 also increases IL2Rα, and this effect can be seen in both murine and human cells, suggesting cross reactivity between mouse IL2 receptors with the the human IL2 in pegIL2 (Figure S3B). To determine the dose of IL2 for mice, we quantified IL2 in serum 6 days post-administration of 4, 8, or 16 ug and found only the 16 μg dose was still detectible at that time point (Figure 3D). To best promote T cell survival, 16 μg pegIL2 was delivered 1 day before lymphocyte toxicity had been detected after ^90^Y-NM600 delivery (Figure 3E). RBCs showed a small but significant reduction on days 6 and 13 after pegIL2 and ^90^Y-NM600 + pegIL2 (Figure 3F). Neutrophils showed no change across any group over the 20 day period. Notably, monocytes were significantly reduced in the ^90^Y-NM600 + pegIL2 group compared to ^90^Y-NM600 on day 20. Lymphocytes decreased at day 6 in both the ^90^Y-NM600 group and the ^90^Y-NM600 + pegIL2 groups relative to PBS control. While lymphocyte levels were no longer depressed by day 13 in the ^90^Y-NM600 + pegIL2 group, those treated with ^90^Y-NM600 alone remained depressed. By day 20 no significant difference in lymphocytes was detectable amongst any treatment group. These data suggest that while toxicity to some immune cell subsets may persist, pegIL2 induced a more rapid recovery of lymphocytes following ^90^Y-NM600. We further observed that ^90^Y-NM600-induced CD8^+^ T cell reduction is increased by pegIL2, which showed increased IL2R-α and -β expressions (Figure 3G).

We next asked whether combined ^90^Y-NM600 + pegIL2 yields a synergistic anti-tumor response in mice bearing MOC2 or SCC7 HNSCC or an unrelated, immunologically cold lung tumor model, Lewis Lung Carcinoma (LLC). Neither ^90^Y-NM600 nor pegIL2 alone decreased tumor growth or survival in any of the three tumor models (Figure 3H-P). In contrast, combined ^90^Y-NM600 + pegIL2 decreased tumor growth and increased survival in all three tumor models compared to PBS alone or any single therapy. Among MOC2 tumor-bearing mice receiving combination ^90^Y-NM600 and pegIL2, 33.3% (3 out of 9) of mice remained tumor-free. These data suggest combining pegIL2 with ^90^Y-NM600 potentiates a synergistic response to control tumor growth that cannot be achieved by either treatment alone.

**Figure 4:**
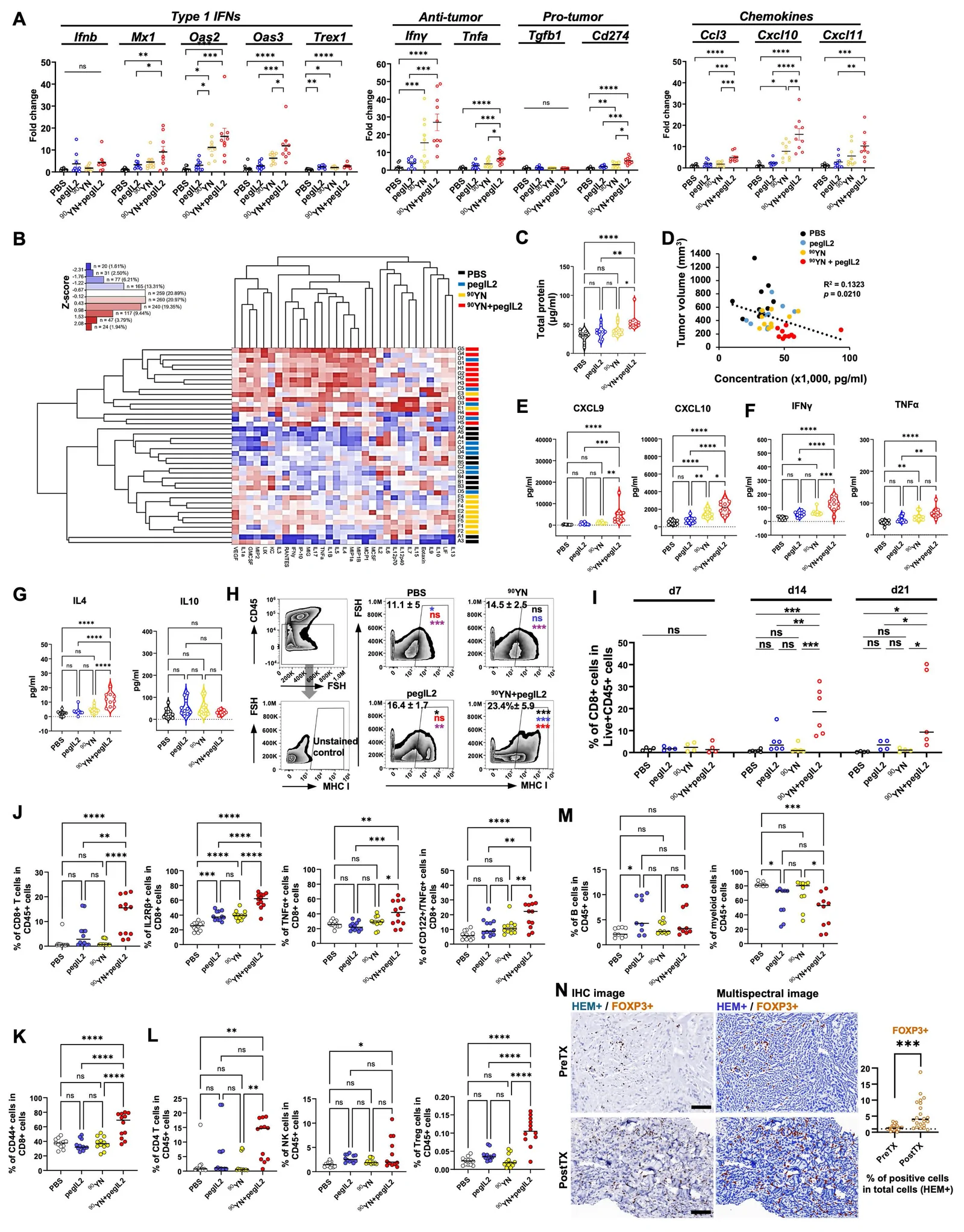
** PegIL2 rescues lymphocyte deficiency following ^90^Y-NM600 and augments anti-tumoral immune responses. A-H**, Mice bearing a MOC2 tumor in the right flank were generated and treated as indicated in Figure 3E. Tumors were collected on day 17, and RNA and protein were extracted from each cohort. (**A**) Relative mRNA expression. (n = 9/group) **B**-**G,** Multiplex analysis of cytokine/chemokine production from tumor. (n = 10/group) (**B**) Heatmap of protein hierarchically clustered by treatment. (**C**) Total protein. (**D**) Correlation between tumor volume and total protein production. (**E**-**G**) Individual cytokine/chemokine concentrations. (**H**) MHC I-expressing tumor (CD45^-^) cells. (n = 10/group) (**I**) Tumor infiltrated CD8^+^ T cells at indicated times. (n = 4-6/group) (**J**-**M**) Tumor-infiltrated immune cells on day 17 post-treatment. (n = 12/group) **(N)** Immunohistochemistry (FOXP3^+^) images from tumor biopsies. HEM; hematoxylin, scale bar is 50 µm. *P* values: ∗ ≤ 0.05; ∗∗ ≤ 0.01; ∗∗∗ ≤ 0.001 by (A-M) one-way ANOVA with Tukey’s correction for multiple comparisons or (N) unpaired T test.

To understand the immune cell response to pegIL2 and ^90^Y-NM600, we analyzed genes associated with the tumor immune response (Figure 4A). Unlike the shorter 7-day time point (Figure 2E), *Ifnb* mRNA showed no change across treatments on day 17 (Figure 4A), whereas, downstream IFNβ target genes, *Mx1*, *Oas2*, *Oas3*, and *Trex1* were increased by ^90^Y-NM600 + pegIL2 compared with the PBS control. Amongst anti-tumor immune cytokines, both *Ifng* and *Tnfa* mRNA were elevated by ^90^Y-NM600 + pegIL2 compared to the PBS control. Among the two pro-tumor immune genes, *Tgfb1* showed no change while *Cd274* (the PDL1 gene) increased after ^90^Y-NM600 + pegIL2 compared with the PBS control. Chemokines *Ccl3*, *Cxcl10*, and *Cxcl11* were elevated by ^90^Y-NM600 + pegIL2 but not by any monotherapy. These changes suggest that combined ^90^Y-NM600 + pegIL2 may affect signaling to immune cells during the recovery period between day thirteen and day twenty, an effect that may not be replicated with monotherapy.

To validate gene expression profiling and to identify effector proteins that augment the anti-tumor response, we measured protein levels of cytokines and chemokines in the tumor (Figure 4B). Unbiased hierarchal clustering based on cytokines and chemokines showed strong distinctions between ^90^Y-NM600 + pegIL2 and monotherapies. The total amount of cytokine and chemokine protein detected was significantly higher in ^90^Y-NM600 + pegIL2 compared to PBS or either monotherapy (Figure 4C), which resulted in a significant inversed proportion between total cytokine and chemokine and tumor volume (Figure 4D). With the exception of CCL2, all chemokines and proinflammatory cytokines were increased by ^90^Y-NM600 + pegIL2 (Figure 4E and S4A-C). Amongst cytokines associated with an anti-tumor immune response, IFN and TNF were elevated after ^90^Y-NM600 + pegIL2 treatment (Figure 4F), consistent with mRNA expression (Figure 4A). Among three cytokines associated with pro-tumor immune signaling (IL4, IL10, and IL13), IL4 was elevated after ^90^Y-NM600 + pegIL2 (Figure 4G).

Given the changes in cytokine and chemokine signaling and lymphocyte recovery after combined ^90^Y-NM600 + pegIL2 treatment, we next examined MHC I expression on day 17. Although the elevated IFNβ levels induced by ^90^Y-NM600 monotherapy on day 7 (Figure 2F) were lost by day 17, elevated MHC I levels persisted to day 17 after combined treatment with ^90^Y-NM600 and pegIL2 (Figure 4H). Given that MHC I recognition by CD8^+^ T cells induces their activation, we quantified CD8^+^ T cell tumor infiltration at days 7, 14, and 21 (Figure 4I). ^90^Y-NM600 + pegIL2 increased CD8^+^ T cell infiltration at day 14, which persisted to day 21. The percent of tumor-infiltrating activated CD8^+^ T cells that were IL2Rβ^+^, TNFα^+^, and double-positive IL2Rβ^+^TNFα^+^ CD8^+^ T cells also increased (Figure 4J, S4D [gating strategy]). IL2Rβ is not only an IL2-uptake receptor but also a memory marker 35. We therefore quantified the CD8^+^ T cell memory marker CD44, finding it, too, was increased by ^90^Y-NM600 + pegIL2 (Figure 4K). Other lymphocytes, CD4^+^ T cells, NK cells, and regulatory T cells (Tregs) also showed increased infiltration with ^90^Y-NM600 + pegIL2 compared with the PBS control (Figure 4L). B-cell infiltration was unchanged, whereas myeloid cell infiltration decreased (Figure 4M). The increased Treg infiltration was reflected in patient tumors from the clinical trial, in which we detected increased Treg infiltration (FOXP3^+^) in post-treatment compared with pre-treatment biopsies (Figure 4N).

We validated the immune infiltration analysis in SCC7 models, which, like MOC2, demonstrated increased CD8^+^ T cell infiltration and decreased myeloid cell infiltration after ^90^Y-NM600 + pegIL2 treatment compared to PBS; in contrast to MOC2, no change in CD4^+^ and Treg cells was detected (Figure S4E). The SCC7 models also replicated the finding that ^90^Y-NM600 + pegIL2 stimulated an increase of IL2Rβ^+^, TNFα^+^, or double IL2Rβ^+^TNFα^+^ CD8^+^ T cells (Figure S4F). These findings suggest that ^90^Y-NM600 + pegIL2 promotes effector/memory CD8^+^ T cell infiltration in preclinical models.

### Combined ^90^Y-NM600 + pegIL2 stimulates tumor-specific memory CD8^+^ T cells and tumor-antagonistic TNFα

**Figure 5:**
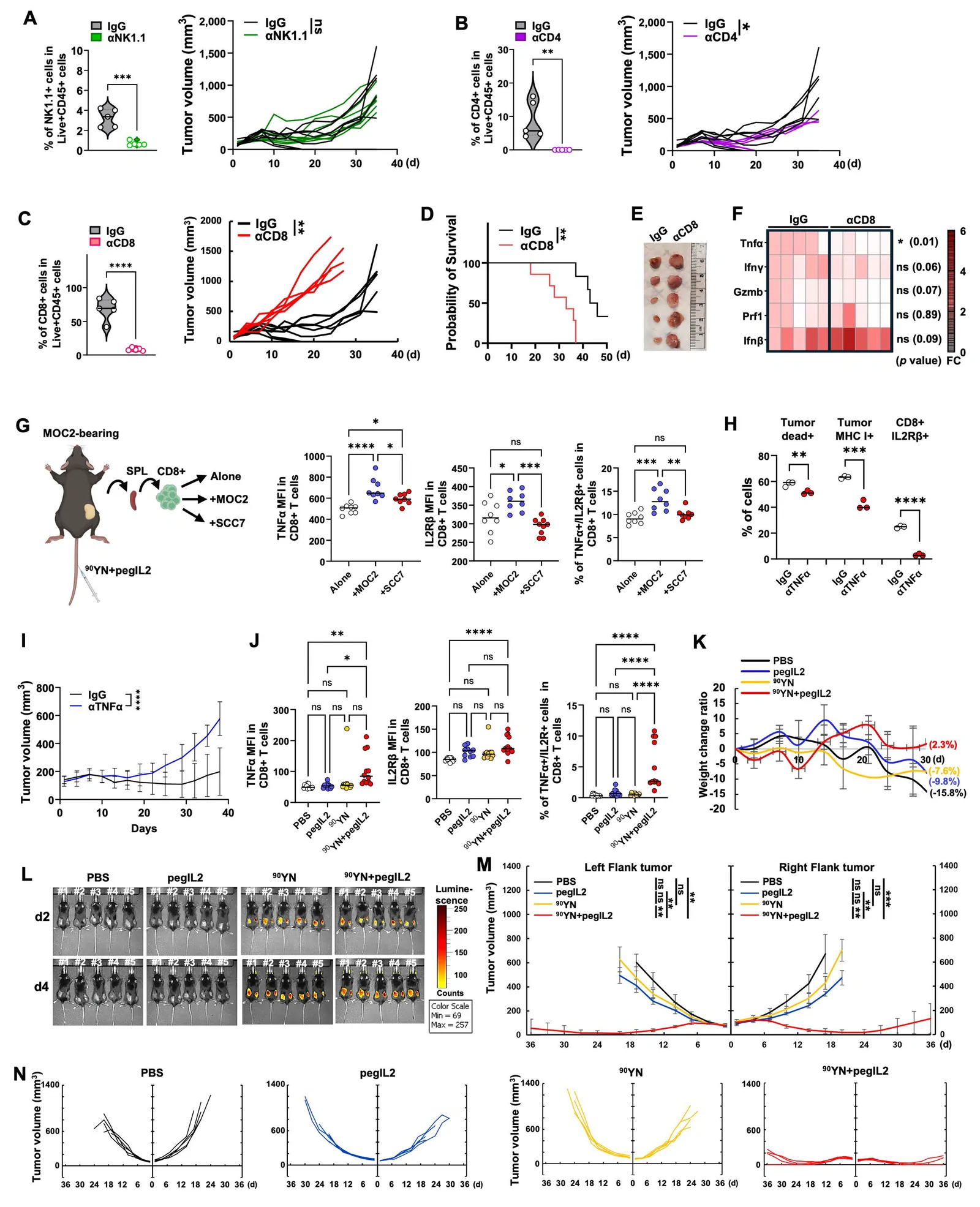
** Combined ^90^Y-NM600 + pegIL2 stimulates tumor-specific memory CD8^+^ T cells and tumor-antagonistic TNFα. A**-**E**, Mice bearing a MOC2 tumor in the right flank were generated and treated with ^90^Y-NM600 and pegIL2, as indicated in Figure 3E. The mice further received IgG or anti-NK, CD4, or CD8 depletion antibody. (n = 5-8/group) (**A**-**C**) Depletion efficacy and tumor volume curves. (**D**) Survival rate. (**E**) Whole tumors. (**F**) Relative mRNA expression analysis of tumors. (n = 5/group) **(G-H)** CD8^+^ T cells were isolated from spleens of mice bearing MOC2 tumors after ^90^Y-NM600 + pegIL2 treatment and were cultured alone or co-cultured with either MOC2 or SCC7 cells. (**G**) TNFα and ILR2β expression on CD8^+^ T cells after co-culture with indicated cells. (n = 8/group) (**H**) Quantification of MOC2 tumor cell death, tumor cell MHCI expression, and CD8^+^ T cell CD122 expression with or without TNFα depletion. (n = 3/group) (**I**) Mice bearing a MOC2 tumor in the right flank were generated and treated with ^90^Y-NM600 and pegIL2, as indicated in Figure 3E. The mice further received anti-IgG or anti-TNFα antibody (200 μg/mouse) intraperitoneally on day 8, 10, and 12. (n = 6/group) and tumor growth was quantified. P values: ∗∗∗∗ ≤ 0.0001 by linear mixed effects model with animal-specific random effects and an autoregressive correlation structure to account for repeated measures over time. **(J)** TNFα and CD122 (IL2Rβ) expression on CD8^+^ T cells isolated from blood of mice in Figure 3E. (n = 10-12/group) (**K**) Body weight over time in bilateral MOC2 -bearing mice. (n = 6-9/group) (**L**) IVIS imaging of bilateral MOC2-bearing mice. (**M**) Mean tumor volume curves. (n = 5/group) (**N**) Individual tumor growth curve. P values: ∗ ≤ 0.05; ∗∗ ≤ 0.01; ∗∗∗ ≤ 0.001 by (A, C, H) two-way T test (G, I) one-way ANOVA with Tukey’s correction for multiple comparisons; (A, B, C, L) growth curves, linear mixed effects model with animal-specific random effects and an autoregressive correlation structure to account for repeated measures over time; (D) Log-rank test. Error bars: Standard deviation.

To determine what immune cells may be required for the anti-tumor response to ^90^Y-NM600 + pegIL2, we depleted NK cells, CD4^+^ T cells, or CD8^+^ T cells in conjunction with ^90^Y-NM600 + pegIL2 therapy. NK cell depletion did not affect tumor growth (Figure 5A). CD4^+^ T cell depletion improved anti-tumor responses (Figure 5B), suggesting that CD4^+^ T cells play a pro-tumor regulatory role, as evidenced by increased Tregs (Figure 4L-4N and Figure S4D), an anti-inflammatory cell lineage derived from CD4^+^ T cells. CD8^+^ T cell depletion resulted in increased tumor growth and reduced mouse survival compared to the non-depleted mice (Figure 5C-D). The final tumor size of CD8-depleted tumor was larger than control (Figure 5E) and had significantly reduced* Tnf* mRNA compared to non-depleted IgG control (Figure 5F). While there was a trend toward reduction by CD8 depletion, no significant difference was detected between CD8-depleted or control tumors among the antitumor cytokines *Ifnγ* and *Granzyme B* (*GzmB*).

To determine antigen-specific T cell memory acquisition, we sorted CD8^+^ T cells from spleens of MOC2-bearing mice 30 days after ^90^Y-NM600 + pegIL2 treatment. These cells were cultured in the absence or presence of either MOC2 or SCC7 (Figure 5G and S5A [gating strategy]). IL2Rβ^+^, TNFα^+^, and IL2Rβ^+^TNFα^+^ CD8^+^ T cells increased when co-cultured with MOC2 (Figure 5G). In contrast, when co-cultured with SCC7, TNFα^+^ CD8^+^ T cells increased only slightly compared to CD8^+^ T cells alone, and this increase was significantly less than found when co-cultured with MOC2. IL2Rβ^+^ and TNFα^+^IL2Rβ^+^ CD8^+^ T cells remained unchanged when CD8^+^ T cells were co-cultured with SCC7. This suggests that tumor-specific memory can develop after ^90^Y-NM600 + pegIL2 treatment and that IL2Rβ and TNFα can serve as markers for tumor-specific memory CD8^+^ T cells in HNSCC. TNFα has been shown to have dual functions, acting as both an inflammatory cytokine that can trigger tumor necrosis or act as a tumor survival and growth factor 36, 37. Given that ^90^Y-NM600 + pegIL2 induces TNFα expression in CD8^+^ T cells after tumor recognition, we examined the role of TNFα in the HNSCC model. Recombinant TNFα dose-dependently stimulated MHC I expression in MOC2 cells, which was reversed by TNFα neutralization (Figure S5B). Blocking TNFα in a co-culture of MOC2 with memory-enriched CD8^+^ T cells isolated from spleens of ^90^Y-NM600 + pegIL2-treated mice reduced tumor cell death, MHC I expression on tumor cells, and IL2Rβ expression in CD8^+^ T cells (Figure 5H and S5C [gating strategy]), indicating TNFα expressed by memory CD8 T cells is acting as an anti-tumor factor. This was reflected *in vivo*, where TNFα blockade significantly increased tumor growth in MOC2 bearing mice treated with ^90^Y-NM600 + pegIL2 (Figure 5I), indicating a role for TNF in the anti-tumor response of this therapeutic regimen.

We next asked whether memory acquisition was also affected in circulating cells. TNFα, IL2Rβ, and double-TNFα^+^IL2Rβ^+^ CD8^+^ T cells increased in blood after treatment with ^90^Y-NM600 + pegIL2, indicating systemic augmentation of CD8^+^ T cell immunity (Figure 5J and S5D [gating strategy]). Given this increase in circulating CD8^+^ T cells, we next examined whether the combination could antagonize multiple tumors in bilateral MOC2-bearing mouse models. In these models, only ^90^Y-NM600 + pegIL2 did not reduce body weight compared with the other treatments (Figure 5K). We detected bilateral tumor targetability of ^90^Y-NM600 using Cherenkov luminescence imaging (Figure 5L) 38. ^90^Y-NM600 + pegIL2 elicited stronger anti-tumor responses than monotherapies in bilateral tumor-bearing mice (Figure 5M-N). To determine whether the treatment contributes to the acquisition of circulating memory CD8^+^ T cells, mice rendered disease-free of MOC2 tumors by ^90^Y-NM600 + pegIL2 were rechallenged with MOC2 cells intravenously followed by quantification of CD62^-^CD44^+^ blood effector memory T cells 3. Effector memory T cells in the blood increased in mice treated with ^90^Y-NM600 + pegIL2 compared to tumor-naïve control mice, indicating systemic memory T cell acquisition against MOC2 (Figure S5E).

Given the resulting blood lymphocyte toxicity from ^90^Y-NM600, we asked whether ^90^Y-NM600 + pegIL2 can expand naïve CD8^+^ T cells in tumor cell co-culture. We isolated CD8^+^ T cells from spleens of tumor-naïve mice and cultured them with or without MOC2 cells. Both pegIL2 and MOC2 cells individually increased live CD8^+^ T cells, but this increase was greatest when both pegIL2 and MOC2 cells were added to naïve CD8^+^ T cell cultures (Figure S5F). Interestingly, in the presence of ^90^Y-NM600, MOC2 cells alone but not pegIL2 alone increased live CD8^+^ T cells, but addition of pegIL2 and MOC2 cells in the presence of ^90^Y-NM600 induced the greatest increase in live CD8^+^ T cells (Figure S5G). The combination of pegIL2 and MOC2 cells in the presence of ^90^Y-NM600 also increased TNFα expression and the proliferation marker Ki-67 in CD8^+^ T cells (Figure S5H). Together, these data suggest that naïve CD8^+^ T cells can be expanded in the tumor microenvironment and activated by ^90^Y-NM600 and pegIL2.

### Cooperative therapeutic interaction of ^90^Y-NM600 + pegIL2 requires STING-mediated IFNβ expression in tumor cells

**Figure 6:**
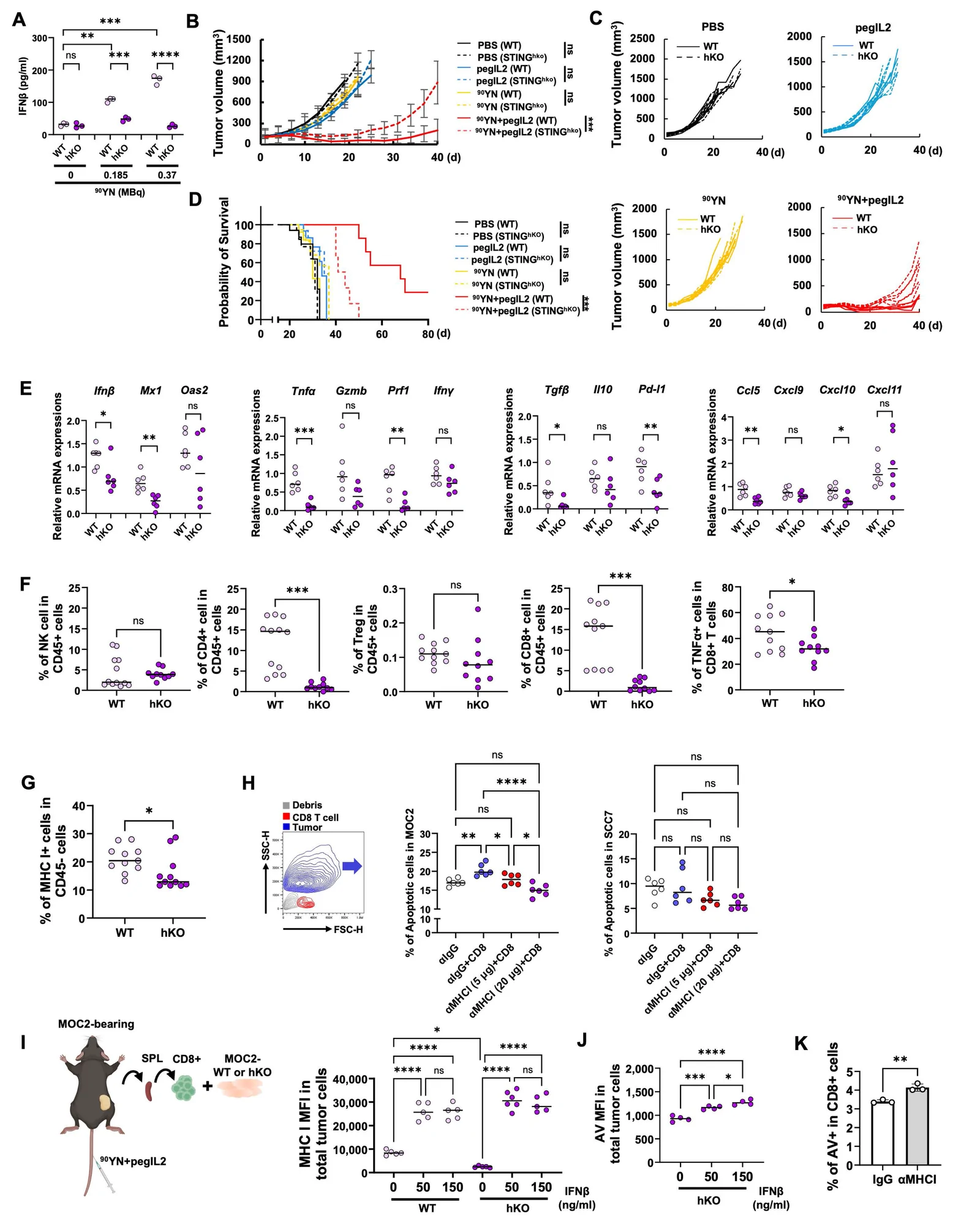
** Cooperative therapeutic interaction of ^90^Y-NM600 + pIL2 requires STING-mediated IFNβ expression in tumor cells.** (**A**) ^90^Y-NM600-induced IFNβ secretome analysis. (n = 3/group) (**B-D**) Mice bearing MOC2-WT or STING^hko^ in the right flank were generated and treated ^90^Y-NM600 and pegIL2 as indicated in Figure 3E. (n = 6/group) (**B**) Mean tumor volume curves. (**C**) Individual tumor growth. (**D**) Survival curves. (**E**-**G)** Tumors collected at day 17 from another cohort of mice treated as in B-D were analyzed. (**E**) Relative mRNA expression in tumor (n = 6/group) (**F**) tumor-infiltrated immune cells (n = 10-12/group). (**G**) MHC I expression on tumor cells. (n = 11/group). (**H**) Quantification of apoptotic MOC2 and SCC7 tumor cells after co-culture with CD8^+^ T cells in the presence or absence of anti-MHCI antibody (αMHCI). (n = 6/group). (**I**) MHC I and (**J**) Annexin V (AV) on tumor cells and **K**) Annexin V (AV) on CD8^+^ T cells after memory CD8^+^ T cell co-culture with MOC2-WT or MOC2-STING^hKO^. (n = 3-6/group) *P* values: ∗ ≤ 0.05; ∗∗ ≤ 0.01; ∗∗∗ ≤ 0.001 by (A, H, I, J) one-way ANOVA with Tukey’s correction for multiple comparisons; (E, F, G, K) unpaired T test; (C) linear mixed effects model with animal-specific random effects and an autoregressive correlation structure to account for repeated measures over time. Error bars: Standard deviation.

We previously reported that immune-susceptibility is stimulated in the TME by EBRT through activation of the STING-IFNβ pathway in murine melanoma models and that STING-heterozygotic knockout (STING-hKO) in HNSCC MOC2 reduced STING expression and activity 2. We therefore investigated here whether RPT in HNSCC works through a STING-IFNβ mechanism. We found that ^90^Y-NM600 increased IFNβ secretion in WT MOC2 cells, and that this increase was suppressed in STING-heterozygotic KO (hKO) MOC2 cells (Figure 6A). While similar tumor growth was observed among STING-hKO-MOC2 and WT-MOC2 tumors when mice were treated with either PBS or pegIL2, the reduced tumor growth induced by ^90^Y-NM600 + pegIL2 was lower in STING-hKO-MOC2 tumors than in WT tumors (Figure 6B-C). This was reflected in reduced survival among mice with STING hKO-MOC2 tumors compared to WT-MOC2 tumors when treated with ^90^Y-NM600 + pegIL2 (Figure 6D). Gene expression changes from tumors collected on day 17 after ^90^Y-NM600 + pegIL2 treatment showed that STING-hKO-MOC2 had reduced mRNA expression related to type I interferon response (*Ifnb1* and *Mx1*), as well as anti-tumor (*Tnf* and *Prf1*) and pro-tumor [*Tgfb1* and *CD274* (Pd-L1)] genes, and the chemokines (*Ccl5* and *Cxcl10*) (Figure 6E).

STING-hKO-MOC2 tumors also had reduced CD4^+^ and CD8^+^ T cell infiltration, with a reduction in TNFα-expressing CD8^+^ T cell infiltration (Figure 6F). Given that STING-hKO-MOC2 tumors have reduced type I interferon signaling and CD8^+^ T cell infiltration, we evaluated tumor MHC I expression, which was significantly reduced in STING-hKO-MOC2 compared to WT tumors (Figure 6G). We also found that an MHC I-blocking antibody suppressed apoptosis amongst MOC2 cells but not SCC7 cells when these tumor cells were co-cultured with memory-enriched T cells isolated from mice bearing WT-MOC2 tumors treated with ^90^Y-NM600 + pegIL2 (Figure 6H). Given that IFNβ plays a role in increasing MHC I in tumor and enabling CD8^+^ T cell surveillance of tumor cells 39, we tested whether IFNβ was required for the CD8^+^ T cell-mediated anti-tumor response. We co-cultured either STING-WT-MOC2 or STING-hKO-MOC2 cells with memory-enriched T cells isolated from mice bearing WT-MOC2 tumors treated with ^90^Y-NM600 + pegIL2 (Figure 6I). STING-hKO-MOC2 co-culture showed reduced MHC I expression compared to WT, but exogenous IFNβ stimulation rescued the reduced MHC I expression in STING-hKO-MOC2 cells, resulting in increased tumor apoptosis by co-cultured CD8^+^ T cells (Figure 6J). Additionally, blocking MHC I increased apoptosis of CD8^+^ T cells during STING-WT-MOC2 co-culture, indicating loss of memory CD8^+^ T cell survival (Figure 6K).

### Immune checkpoint inhibitor promotes anti-tumor immune response with ^90^Y-NM600 + pegIL2

**Figure 7:**
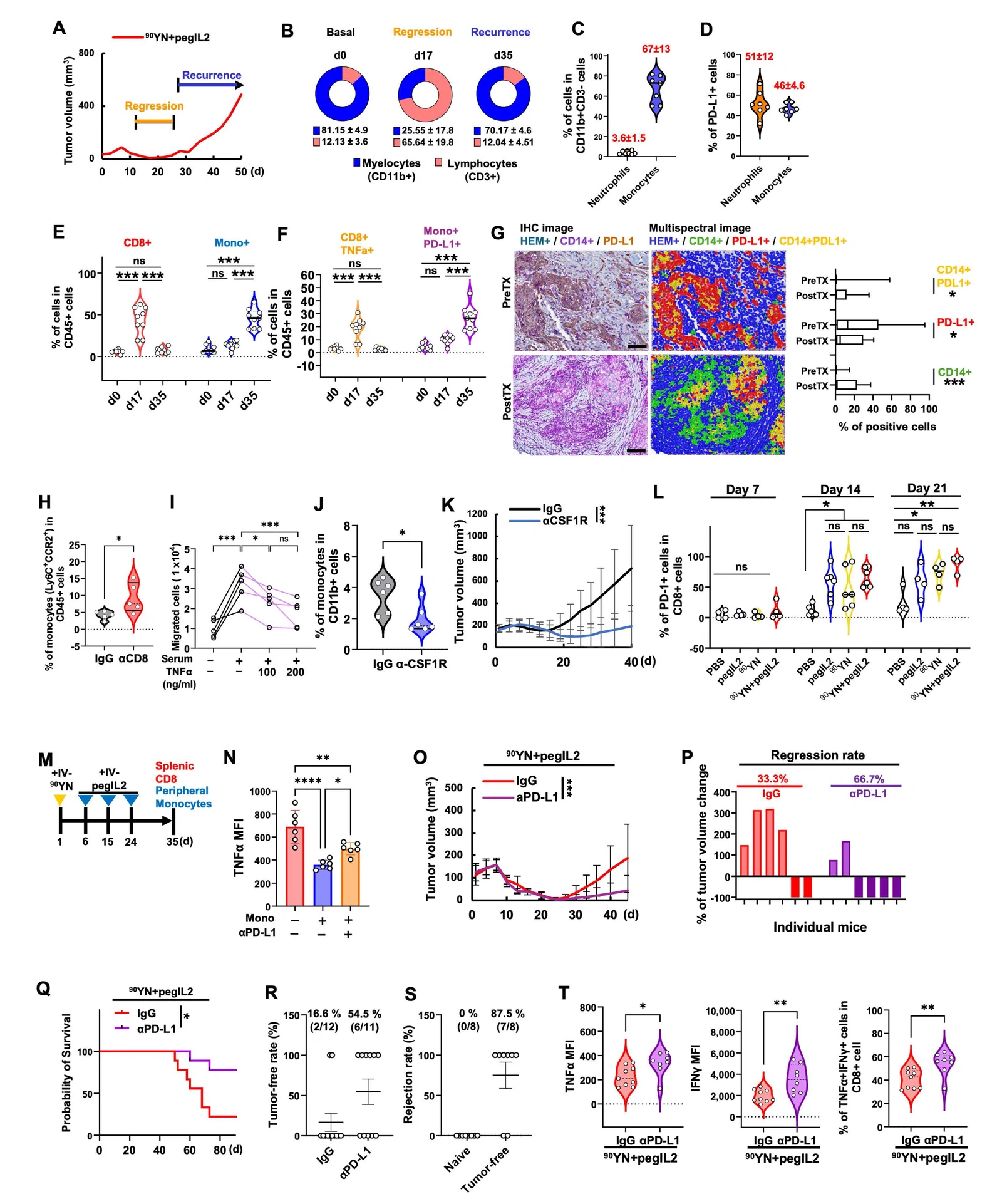
** Anti-PD-1 immune checkpoint inhibition promotes anti-tumor immune response of ^90^Y-NM600 + pegIL2.** (**A**) Tumor volume, pooled from the ^90^Y-NM600 + pegIL2-treated group of Figure 3H. (**B**) Proportion of myelocytes (CD11b^+^ cells) and lymphocytes (CD3^+^ cells) during regression (d17) and recurrence (d35) phases of tumor growth of (n = 5-7/group) (**C**) Tumor infiltrated neutrophils and monocytes at d35. (n = 7/group) (**D**) PD-L1-expressing neutrophils and monocytes at d35. (n = 7/group) (**E**) Tumor-infiltrated CD8^+^ T cells and monocytes over time. (n = 8-9/group) (**F**) TNFα^+^CD8^+^ T cells and PD-L1^+^monocytes over time. (n = 8-9/group) (**G**) Multispectral immunohistochemistry analysis (CD14^+^, PD-L1^+^) in submandibular mass (left) and quantification of cell types from 3 patients (right). HEM; hematoxylin, scale bar is 50 µm. (**H**) Tumor-infiltrated monocytes on day 17. (n = 5/group) (**I**) Monocyte migration ability upon serum and TNFα stimulation. (n = 5/group) **(J)** Monocyte depletion efficacy. (n = 6/group) (**K**) Mean tumor volume curves. (n = 10/group) (**L**) Tumor-infiltrated PD-1^+^CD8^+^ T cells from MOC2-bearing mice at indicated times. (n = 4-6/group) (**M**) Experimental scheme. (**N**) TNFα MFI in CD8^+^ T cells after co-culturing with monocytes. (n = 6/group) **O**-**T**, Mice bearing MOC2 tumors were treated with IgG or anti-PD-L1 during ^90^Y-NM600 + pegIL2 treatment as indicated in Figure 3E. (**O**) Mean tumor volume curves. (n = 6/group) (**P**) Tumor regression rate. (n = 6/group) (**Q**) Survival rate. (n = 9/group) (**R**) Tumor-free rate. (**S**) Rejection rate (complete responses). (**T**) TNFα and IFNγ expression in tumor-infiltrated CD8^+^ T cells at d35. (n = 8-9/group) *P* values: ∗ ≤ 0.05; ∗∗ ≤ 0.01; ∗∗∗ ≤ 0.001 by (E, F, L, N) one-way ANOVA with Tukey’s correction for multiple comparisons; (G, H, T) unpaired T test; (K, O) linear mixed effects model with animal-specific random effects and an autoregressive correlation structure to account for repeated measures over time; (Q) Log-rank test. (R, S) Error bars: Standard error of the mean.

Tumor regression from ^90^Y-NM600 + pegIL2 treatment persisted until day 25, but recurrence occurred around 30 days post-treatment (Figure 7A; referred from Figure 3H). Tumor regression was accompanied by decreased myeloid infiltration at day 17 (Figure 4M) and decreased monocytes in the blood at day 20 (Figure 3F), while CD8^+^ T cell tumor infiltration was elevated up to day 21 (Figure 4I). We hypothesized that dynamic changes in immune cell ratios induced by ^90^Y-NM600 + pegIL2 may drive tumor regression or recurrence and therefore extended our examination of immune cells to day 35 during tumor recurrence. Lymphocyte infiltration increased during the regression stage (day 17) but decreased during the recurrence stage (day 35) (Figure 7B and S6A [gating strategy]). In contrast, myeloid cell infiltration increased during the recurrence stage, with tumor-infiltrated myeloid cells composed mainly of monocytes (67%) and with few neutrophils (3.6%) (Figure 7C). These tumor-infiltrated monocytes and neutrophils expressed PD-L1 in the recurrent stage of the tumor (Figure 7D). Pro-tumor myeloid cells have been associated with poor outcomes and increased tumor immunosuppression 31. During tumor regression, CD8^+^ T cell infiltration was elevated while monocytes remained unchanged, which was reversed during the recurrence stage, where decreased CD8^+^ T cell infiltration was accompanied by increased monocyte infiltration (Figure 7E).

Given the decrease in *Tnf* expression accompanying CD8^+^ T cell depletion (Figure 5F) and that TNFα is expressed by tumor-recognizing CD8^+^ T cells (Figure 5G), we hypothesized that TNFα secreted by CD8^+^ T cells has an antagonist role in monocyte infiltration. Similar to the change of CD8^+^ T cell and monocyte infiltration ratio, the TNFα-expressing CD8^+^ T cell and PD-L1-expressing monocyte ratio was changed from d17 to d35 (Figure 7F). Notably, the increased TNFα levels in serum seen with ^90^Y-NM600 + pegIL2 treatment at d17 returned to baseline by day 35 (Figure S6B). In the phase II clinical trial, we also observed that monocytes expressing PD-L1 (CD14^+^PD-L1^+^) increased PostTX compared to PreTX (Figure 7G). In the murine model, CD8^+^ T-cell depletion yielded increased monocyte infiltration (Figure 7H). Based on these observations, on day 35 when monocyte infiltration was dominant, we sorted monocytes from PBMCs and collected serum to assess the role of TNF in monocyte migration. In the presence of day-35 serum, the number of migrated monocytes significantly increased; however, TNFα added to the serum inhibited that increased migration, suggesting that TNFα-expressing memory CD8^+^ T cells could play a role in suppressing myeloid cell infiltration in the TME (Figure 7I).

To determine whether monocyte depletion could prolong anti-tumor responses from ^90^Y-NM600 + pegIL2, we quantified tumor volume after treatment with the monocyte-depleting anti-CSF1R antibody and found that monocyte deficiency strongly suppressed tumor growth (Figure 7J-K). We next asked whether an anti-PD-L1 immune checkpoint inhibitor could improve anti-tumor responses with ^90^Y-NM600 + pegIL2. When MOC2-tumor bearing mice are treated with ^90^Y-NM600 + pegIL2, tumor-infiltrating CD8^+^ T cells have increased PD-1 expression by day 21 (Figure 7L). To determine if increasing PD-L1 expression limits CD8^+^ T-cell immunity when interacting with monocytes, we cultured peripheral monocyte and splenic CD8^+^ T cells isolated from ^90^Y-NM600 + pegIL2-treated mice with pre-plated MOC2 (Figure 7M). Monocytes inhibited TNFα expression in CD8^+^ T cells but expression was partially rescued by anti-PD-L1 antibody treatment (Figure 7N). In mice bearing MOC2 tumors, addition of anti-PD-L1 antibody therapy to ^90^Y-NM600 + pegIL2 led to a longer delay in tumor growth after the initial tumor regression and higher regression rate (Figure 7O-P). Addition of anti-PD-L1 also increased mouse survival (Figure 7Q), with a striking 54.5% of mice tumor-free at day 90 and an 87.5% tumor-rechallenge rejection rate (Figure 7R-S). In a separate cohort of mice, we collected tumors at day 35 and detected elevated TNFα and IFNγ expression in the infiltrated CD8^+^ T cells, as well as increased infiltration of polyfunctional CD8^+^ T cells (double TNFα^+^IFNγ^+^) with addition of anti-PD-L1, suggesting that blocking PD-L1 increases T-cell immunity when combined with ^90^Y-NM600 and pegIL2 (Figure 7T, S6C [gating strategy]).

## Conclusions

The switch from immunologically “cold” to “hot” tumors is an important goal in immunotherapy, and various therapy combinations have been attempted to achieve this. While a clinical study of focal EBRT + ICI in metastatic cancer showed no benefits compared to ICI alone in patients with HNSCC 6, we speculated based on prior preclinical data 19 that with additional immune stimulation we might identify a therapeutic synergy between RT and ICI. Although the ability of IL2 to promote proliferation and activation of T cells provides a potential avenue for such amplification 3, 40, its drawbacks include a short half-life, severe side effects, and stimulation of Treg cells.

Thus, we combined pegIL2, EBRT, and pembrolizumab in a phase II clinical trial that was terminated early due to a manufacturer’s decision to discontinue all clinical investigations of pegIL2. No statistically significant inferences can be drawn from the clinical trial because of its small sample size. However, the data obtained prior to study termination are notable in suggesting that combined pegIL2, RT, and pembrolizumab are tolerable in patients. The ORR with the combination therapy was 40% in all patients. While there was one death unrelated to treatment, the remaining four patients had reduced tumor size after treatment, with two of those patients meeting the criteria for clinical benefit. The limited accrual of this phase II clinical trial and its single-arm design do not enable determination of whether clinical responses, including ORR, PFS, and OS, reflect therapeutic efficacy and the clinical findings presented here can be used for hypothesis generation only. However, despite the manufacturer’s discontinuation of the bempegaldesleukin formulation of pegIL2 that limits translation to the clinic of this formulation, our findings suggest opportunity for continued research into such agents in combination with RT and ICIs. These findings also suggest that the RT combination can mitigate the therapeutic limitations associated with previous pegIL2 and ICI combinations 14.

We observed increased Treg infiltration in both mice and human HNSCC following combinations of RT therapy, pegIL2, and anti-PD-1 therapy. Tregs increased in tumor biopsies after combination treatment, consistent with previous findings from the PIVOT-02 clinical study, which reported increased Treg numbers in the blood after pegIL2 + anti-PD-1 13. Other slow-release forms of IL2 are under investigation, including TransCon IL2/ (Ascendis), a methoxy polyethylene glycol-conjugated IL2 in Phase I/II clinical trials (NCT05081609) 41. Because not all extended-life IL2 will result in similar reductions in Treg stimulation relative to that seen with the bempegaldesleukin formulation of pegIL2 12, careful consideration of design and immune cell interactions is required. PegIL2 that lacks preferential binding of IL2Rβ may increase Tregs and CD8^+^ T cell exhaustion 42. Although we report here increased Treg numbers, the coexistence of CD8^+^ T cells in the presence of Tregs suggested the need for investigation into the effector functionality. Our live single-cell proteomics approach found that the effector:regulatory and chemoattractive:regulatory ratios significantly increased 23 days (C3D1) after RT with two cycles of pegIL2 and pembrolizumab treatment. The tumor biopsies also showed increased IL2Rβ (CD122)-expressing CD8^+^ T cells PostTx, suggesting increased effector CD8^+^ T cell functionality even in the presence of Treg expansion. As noted, tumor RT increased tumor infiltration by effector CD8^+^ T cells in patients. RPT-mediated tumor radiation combined with pegIL2 in murine models similarly promoted tumor infiltration and activation of effector CD8^+^ T cells, along with Treg infiltration in tumor. CD8^+^ T cells were critical to tumor response in murine models, and RT-mediated MHC I induction in the tumor may be a key factor in augmenting CD8^+^ T cell responses, potentially overcoming the limited efficacy observed in the previous PIVOT clinical trial by pegIL2 + anti-PD-1 without RT 14.

Although both EBRT and RPT can deliver radiation to the tumor, RPT differs from EBRT in that radiation is delivered systemically, conferring risk for blood cell toxicity before it reaches the tumor. We previously observed that ^90^Y-NM600 RPT showed no tissue toxicity in the liver, kidney, spleen, and bone marrow 22, and here found no histological signs of liver toxicity 25 days after administration of ^90^Y-NM600; however, potential for liver toxicity may exist, as we have observed signs of late toxicity after delivery of ^225^Ac-NM600 43. In contrast, dose-dependent blood cell toxicity has been observed in a murine melanoma model 22. However, a previous report showed that up to 9.25 MBq of ^90^Y-NM600 was below the maximum tolerable activity (MTA), and blood cell recovery steadily increases over 3 weeks after administration of 4.63 or 9.25 MBq in naïve, tumor-free mice 21. Consistently, we observed blood cell toxicity in HNSCC models, including lymphocyte number, was recovered in week 3 after administration of 3.7 MBq ^90^Y-NM600, which is below the MTA dose that shows optimal tumor MHC I and IFNβ induction. The treatment strategy using the pegIL2 combination overcame the reduction in CD8^+^ T cell numbers induced by ^90^Y-NM600 to levels comparable to those of the untreated control group. Although EBRT and RPT differ in their delivery methods, both tumor RT modalities induced infiltration and activation of effector CD8^+^ T cells in the TME when pegIL2 was present, demonstrating their capacity to be used as parallel strategies. Given that RPT has the benefit of targeting multiple tumor sites, we observed tumor growth suppression in the bilateral tumor model (Figure 5M).

Our preclinical findings presented here and those previously reported 19 are also consistent with a mechanism in which STING activation increases IFNβ secretion, which may stimulate anti-tumor immune response and improve survival. In patients with HNSCC, low STING expression is associated with worse progression free survival 44, 45 and higher STING expression is associated with greater activation of CD4^+^/CD8^+^ T cell interactions 46. We speculate that induction of STING expression or of IFN secretion could serve as potential biomarkers for predicting response or clinical benefit with combination therapeutics that employ low dose RPT to overcome low STING expression in HNSCC.

Tumor recurrence following treatment with RPT and pegIL2 was accompanied by a decrease in TNFα. Serum collected near the time of tumor recurrence stimulated monocyte migration, and adding TNFα to this serum inhibited that migration. Monocyte count remained relatively low in the blood through week 3 after RPT, a period of tumor suppression after the pegIL2 + ^90^Y-NM600 combination. Monocytes that migrate into tumors can have pro-tumor impacts 47-49, and PD-L1-expressing monocytes show an inverse correlation with overall survival 50. Given that the RT + pegIL2 combination increased chemokine levels in mouse tumor models and in CD8^+^ T cells from patients, it is plausible that treatment induced changes in the TME would promote monocyte migration 51, 52. A previous study reported that TNFα can increase PD-L1 expression in monocytes 53. These observations raised the possibility that TNFα-expressing memory CD8^+^ T cells stimulate regulatory monocyte (PD-L1^+^) differentiation and can also inhibit monocyte migration 54. This supports a mechanism for therapeutic benefit from the addition of anti-PD-L1 therapy to pegIL2 + ^90^Y-NM600, which can mitigate these monocyte effects and account for the increased survival and immune memory observed in our mouse models with this triple combination.

As previous studies have identified sensitivity of combination therapies to sequencing of delivery, particularly with regards to radiotherapy/immunotherapy combinations 16, future studies examining the optimal timing of these therapies to maximize immune response and reduce toxicity will be valuable. RPT therapies commonly induce lymphopenia and one potentially critical role of pegIL2 in this study may be in promoting survival and proliferation of lymphocytes in the presence of systemic radiotherapy. Conversely, the finding that tumor-recognizing T cells increase IL2Rβ expression following RT suggests a mechanism whereby RPT may potentiate the response of T cells to pegIL2 or other IL2Rβ agonists. These mechanisms would suggest a potential advantage to delivering pegIL2 or similar such agents after RPT, although the effects of such treatment sequencing and optimization of timing remain to be clarified. Optimizing such parameters may be a key factor in overcoming the lack of improvement over anti-PD-1 therapy alone in the recent Phase III trial of bempegaldesluekin + anti-PD-1 14.

Given that EBRT is limited in its ability to treat metastatic sites, exploring mechanistic interactions of pegIL2 and RPT may support future studies in metastatic disease. Our findings suggest a model in which pegIL2 overcomes CD8^+^ T-cell deficits that result from RPT, and RPT promotes expression of the pegIL2 receptor on CD8^+^ T cells. The combination of these effects promote CD8^+^ T cell infiltration and activation in the radiated TME, a process that is supported by combination with anti-PD-1 for prevention of monocyte-mediated T cell exhaustion. Our preclinical findings here with combinations of RPT, pegIL2, and anti-PD-1 therapy compare favorably with our prior studies of EBRT in situ vaccine approaches in combination with immune checkpoint inhibition 28 and with those combining RPT with checkpoint inhibition 22. Given the distinct mechanistic contributions identified, we next aim to advance early phase clinical studies that build upon our approach here or, in the absence of pegIL2, to combine in situ vaccination, RPT, and immune checkpoint inhibition to prime, propagate, and sustain anti-tumor immune response, respectively.

## Supplementary Material

Supplementary figures and tables.

## Data Availability

All data supporting the findings of this study are available within the paper and upon request.

## Abbreviations

AE: Adverse event
ANOVA: Analysis of variance
AV: Annexin V
BEMPEG: Bempegaldesleukin (NKTR-214)
CBC: Complete blood count
CD25: Interleukin-2 receptor alpha
CD122: Interleukin-2 receptor beta
C1D1: Cycle 1, Day 1
C1D8: Cycle 1, Day 8
C2D8: Cycle 2, Day 8
C3D1: Cycle 3, Day 1
C3D8: Cycle 3, Day 8
C:R: Chemoattractive:Regulatory ratio
CT: Computed tomography
DAB: 3,3′-Diaminobenzidine
DMEM: Dulbecco's Modified Eagle Medium
DMSO: Dimethyl sulfoxide
DOTA: 1,4,7,10-Tetraazacyclododecane-1,4,7,10-tetraacetic acid
EORTC-QLQ: European Organization for Research and Treatment of Cancer Quality of Life Questionnaire
E:R: Effector:Regulatory ratio
EBRT: External beam radiotherapy
ELISA: Enzyme-linked immunosorbent assay
FBS: Fetal bovine serum
FFPE: Formalin-fixed paraffin-embedded
FOXP3: Forkhead box P3
HCl: Hydrochloric acid
hKO: Heterozygous knockout
HNSCC: Head and neck squamous cell carcinoma
HPRT: Hypoxanthine phosphoribosyltransferase
ICI: Immune checkpoint inhibitor
IFNβ: Interferon beta
IFNγ: Interferon gamma
IHC: Immunohistochemistry
IL2: Interleukin-2
IL2Rα: Interleukin-2 receptor alpha
IL2Rβ: Interleukin-2 receptor beta
IRB: Institutional Review Board
Ki-67: Marker of cellular proliferation
LLC: Lewis lung carcinoma
MBq: Megabecquerel
MFI: Median fluorescence intensity
MHC I: Major histocompatibility complex class I
MOC2: Mouse oral carcinoma 2
NaOAc: Sodium acetate
NK: Natural killer
ORR: Objective response rate
OS: Overall survival
PBS: Phosphate-buffered saline
PBMC: Peripheral blood mononuclear cell
PD-1: Programmed cell death protein 1
PD-L1: Programmed death-ligand 1
pegIL2: PEGylated interleukin-2
PET/CT: Positron emission tomography/computed tomography
PFS: Progression-free survival
PostTx: Post-treatment
PreTx: Pre-treatment
PR: Partial response
PSI: Polyfunctionality Strength Index
qPCR: Quantitative polymerase chain reaction
RBC: Red blood cell
RECIST: Response Evaluation Criteria in Solid Tumors
R/M: Recurrent/metastatic
RPMI 1640: Roswell Park Memorial Institute 1640 medium
RPT: Radiopharmaceutical therapy
RT: Radiotherapy
S:R: Stimulatory:Regulatory ratio
SCC7: Squamous cell carcinoma 7
SD: Stable disease, standard deviation
STING: Stimulator of interferon genes
Teff: Effector T cell
TME: Tumor microenvironment
TNFα: Tumor necrosis factor alpha
Treg: Regulatory T cell
t-SNE: t-distributed stochastic neighbor embedding
UWCCC: University of Wisconsin Carbone Cancer Center
WBC: White blood cell
WT: Wild type
Y-NM600: Yttrium-labeled NM600 radiopharmaceutical (e.g., ^86^Y-NM600, ^90^Y-NM600)
