# Supplementary material for: PEGylated IL2 and tumor-targeted radiotherapy augment CD8+ T cell-mediated anti-tumor response to anti-PD-1 in head and neck squamous cell carcinoma
Source: Theranostics. 2026 Aug 12;16(15):8733–56. doi: 10.7150/thno.135255 (PMC13549225; doi:10.7150/thno.135255)
Supplement: Supplementary file 1 [file thnov16p8733s1.pdf]

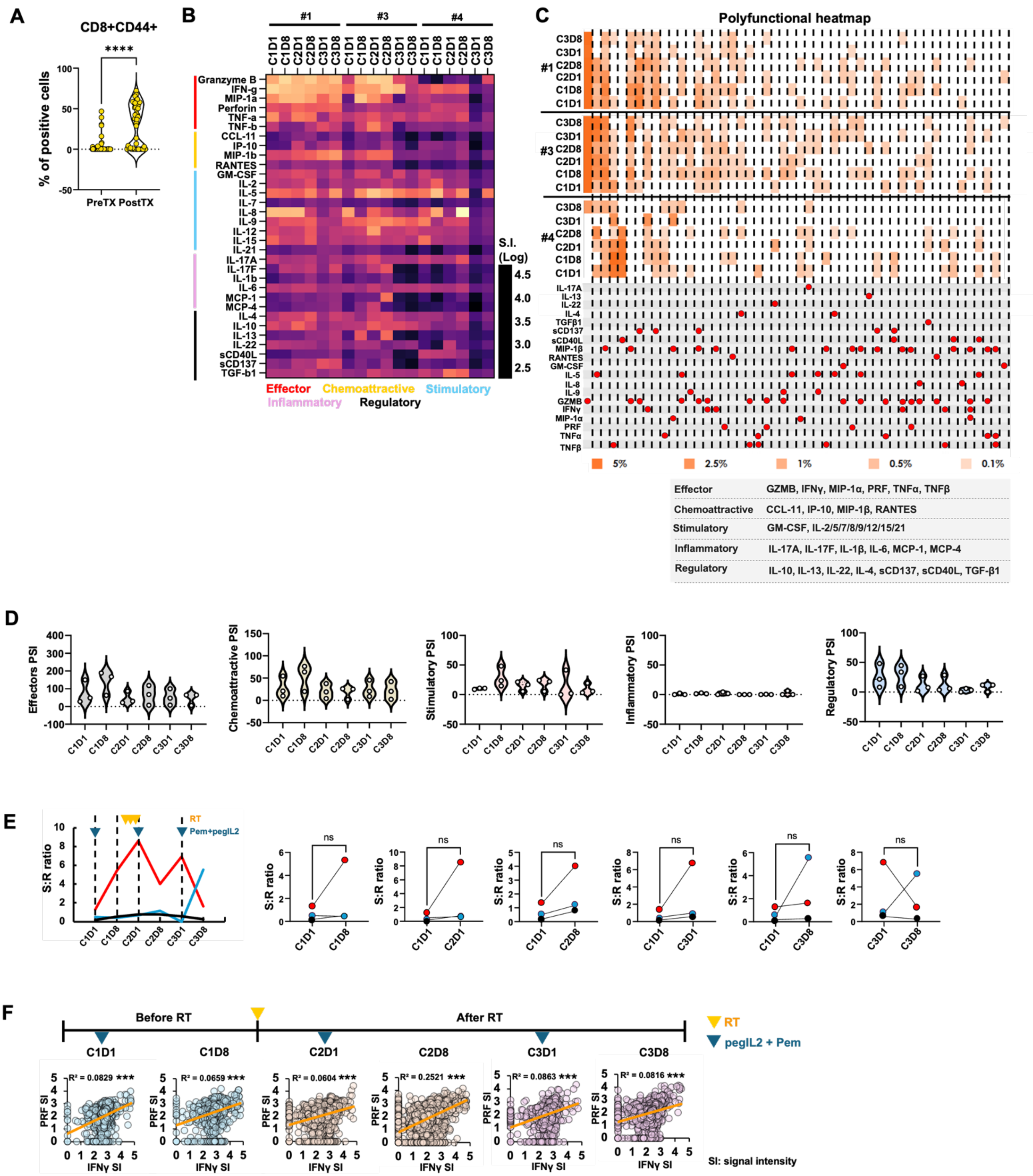

Figure S1. Single-cell proteomics to assess $CD8^+$ T cell functionality in patients with recurrent or metastatic HNSCC patients. (A) Multispectral immunohistochemistry analysis of memory T cells ($CD8^+CD44^+$) infiltration. *P* values: ∗∗∗∗ ≤ 0.0001 by unpaired T test,. **B-D,** $Live^+CD8^+$ T cells sorted from patients' peripheral blood mononuclear cells (PBMCs) were subjected to single-cell multiplex secretome analysis to screen treatment-induced phenotype changes in $CD8^+$ T cells. (**B**) Signal intensity (S.I.) heat map from detected protein. (**C**) Polyfunctional heat map showing single- and multi-protein expression of single cells in each patient at each treatment cycle. **(D)** Polyfunctionality Strength Index (PSI) from each

cycle of each CD8 T cell subset. (**E**) Stimulatory:regulatory (S:R) ratio from each cycle. *P* values: ns, not significant by paired T test (**F**) Correlation within single cells of expression level of indicated proteins. *P* values: $*** \leq 0.001$by two-tailed test.

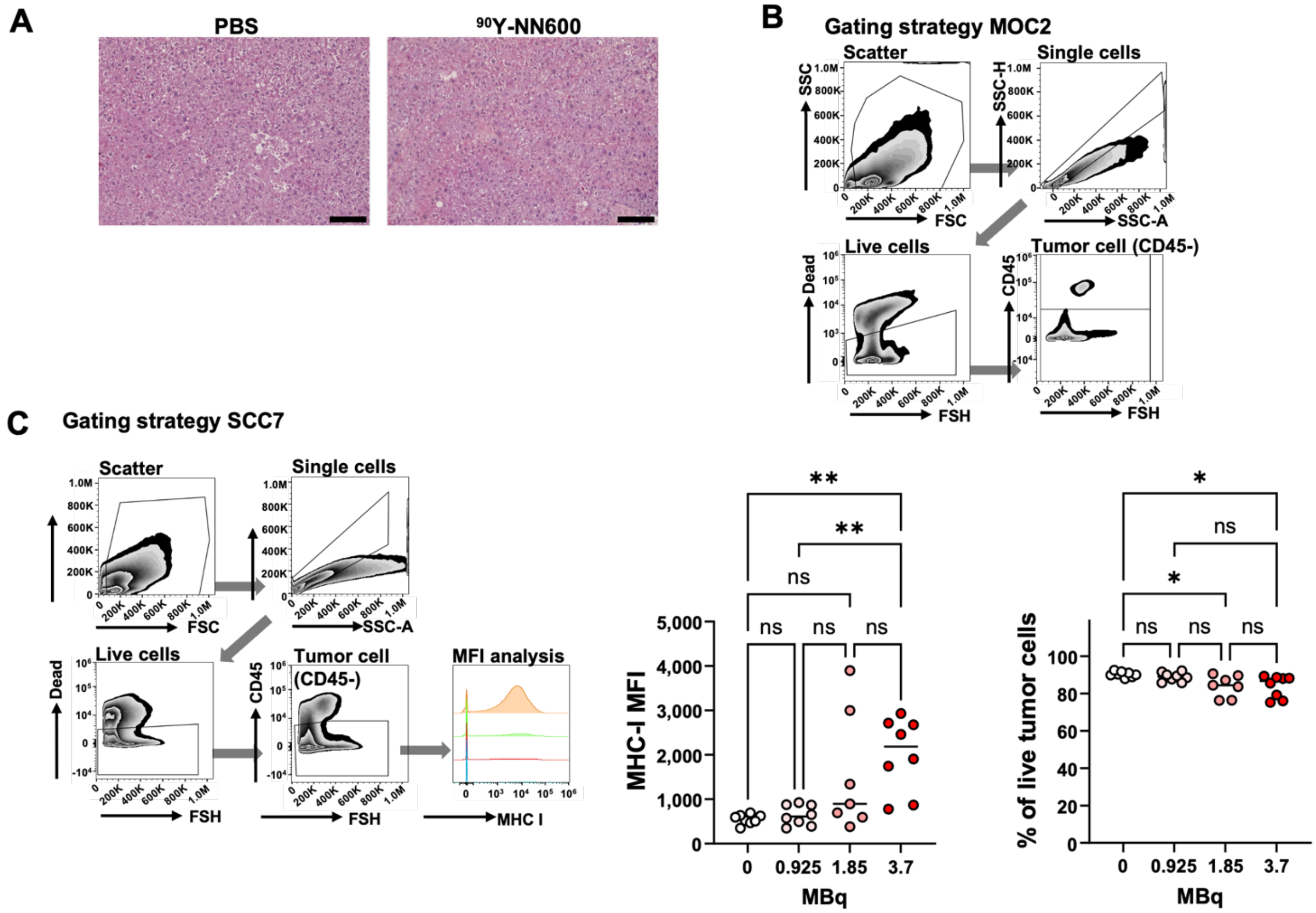

Figure S2. (**A**) C57Bl/6 mice with MOC2 tumors were treated with PBS or ^{90}Y-NN600 on day 1. Fixed livers were H&E stained on day 25 post ^{90}Y-NN600 administration. Scale bar 50 µm. (**B**) Flow cytometry gating strategy (left) and MHC I and live^{+}tumor cell (CD45^{-}) analysis in SCC7 tumor (right). (n=7-8/group) *P* values: $* \leq 0.05$; $** \leq 0.01$; ns, not significant by one-way ANOVA with Tukey's correction for multiple comparisons.

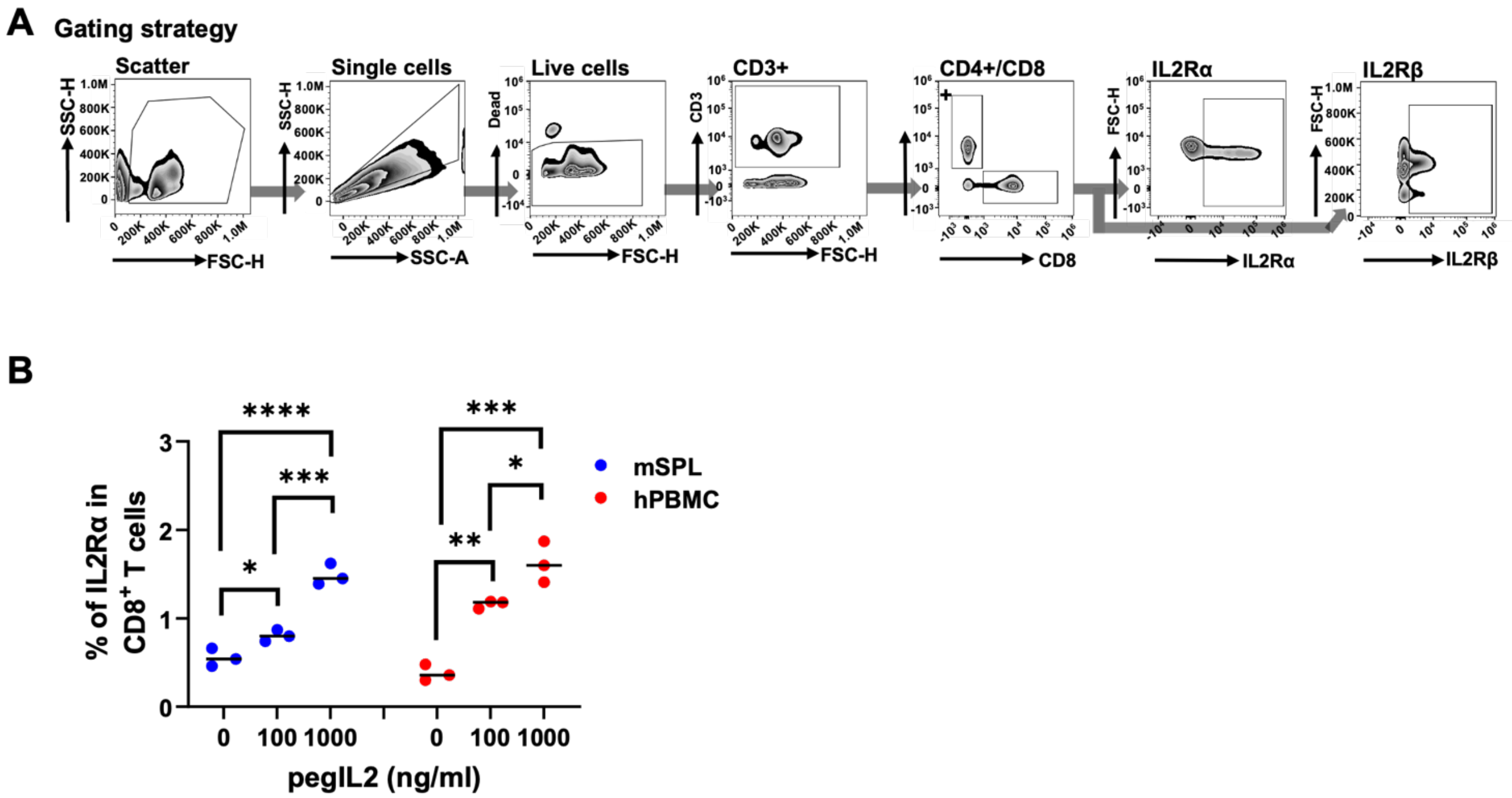

Figure S3. (**A**) Flow cytometry gating strategy. (**B**) Healthy human donor PBMCs (hPBMC) and naïve mouse splenocytes (mSPL) were cultured in the presence of pegIL2 for 72h, then IL2Rα-expressing CD8+ T cells were quantified. *P* values: $* \leq 0.05$; $** \leq 0.01$; $*** \leq 0.001$, $**** \leq 0.0001$ by one-way ANOVA with Tukey's correction for multiple comparisons.

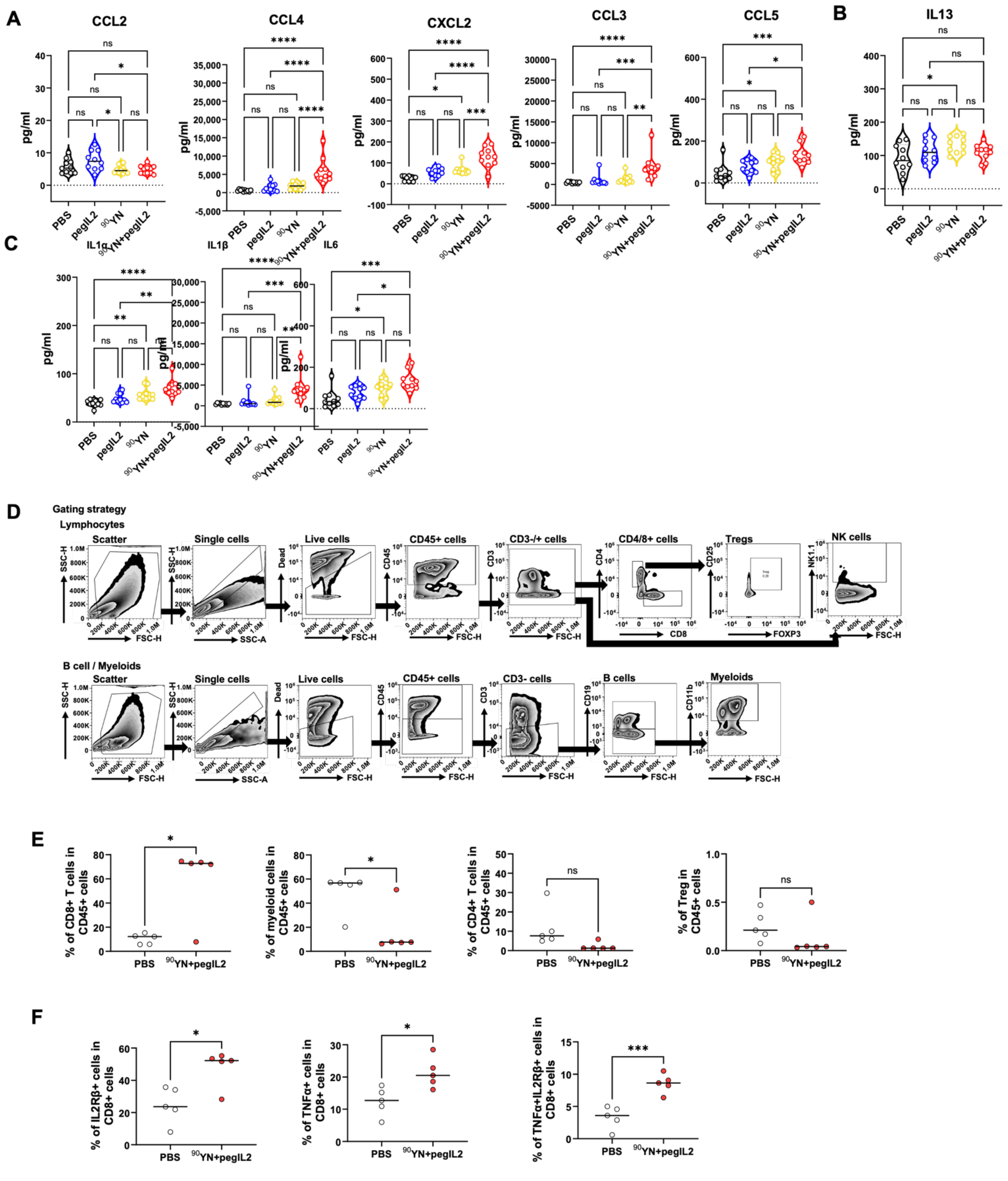

Figure S4. (**A**) Cytokine and (**B-C**) chemokine concentration from tumor multiplex analysis. (n=10/group) *P* values: $* \leq 0.05$; $** \leq 0.01$; $*** \leq 0.001$; ns: not significant by one-way ANOVA with Tukey's correction for multiple comparisons. (**D**) Flow cytometry gating strategy. (**E**) Tumor-infiltrated immune cells and (**F**) IL2Rβ and TNFα expression on $CD8^{+}$ T cells at day 17 post-treatment in SCC7-bearing mice. (n=5/group), *P* values: $* \leq 0.05$; ns: not significant by unpaired T-test.

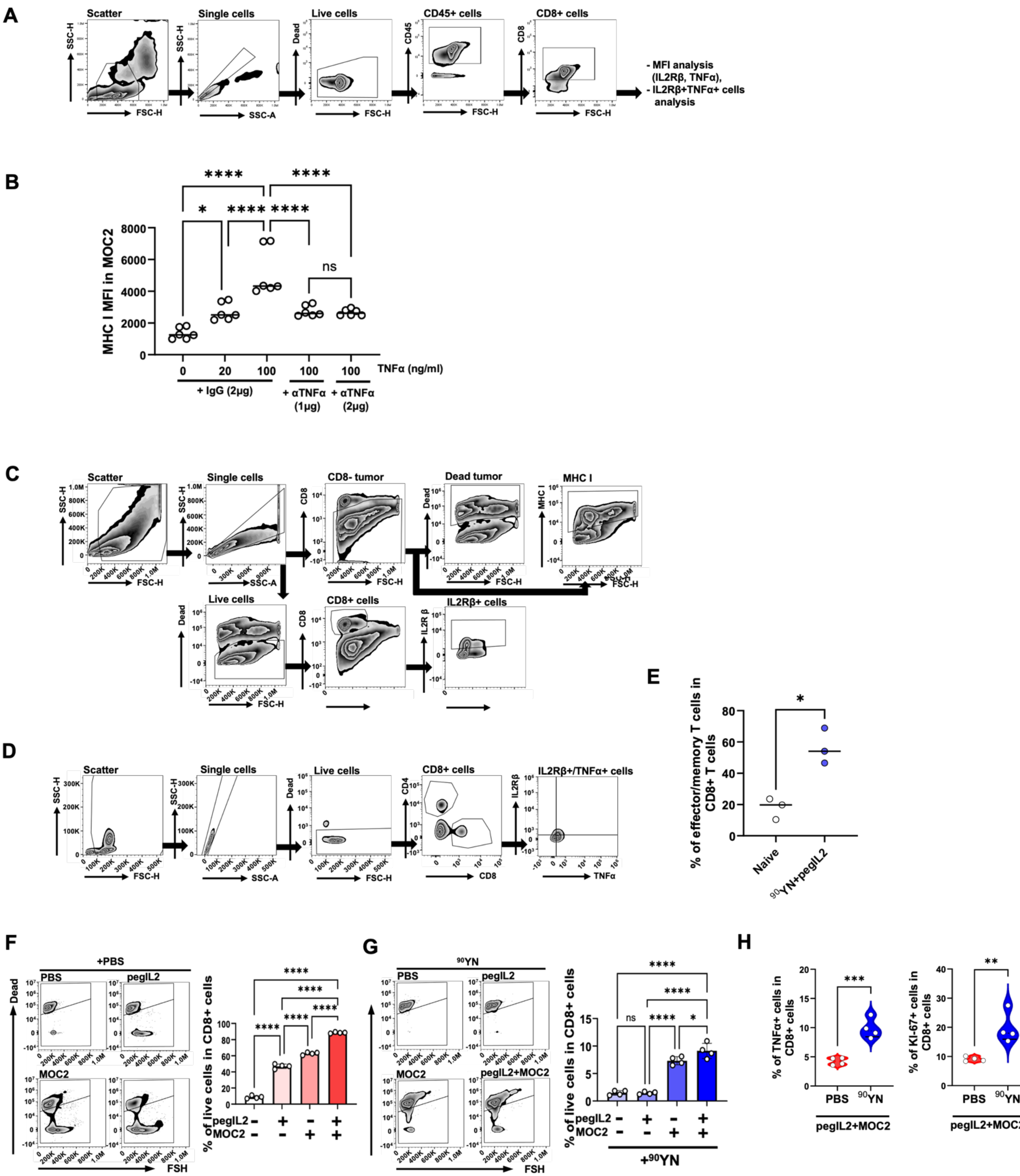

Figure S5. (**A**) Flow cytometry gating strategy. (**B**) Validating recombinant mouse TNFα and anti-TNFα antibody efficacy on MHC I expression in MOC cells. *P* values: $* \leq 0.05$; $**** \leq 0.0001$ by one-way ANOVA with Tukey's correction for multiple comparisons (**C** and **D**) Flow cytometry gating strategy. (**E**) Mice rendered tumor-free by ^{90}Y-NM600 + pegIL2 or control tumor-naïve mice were injected with MOC2 intravenously. Blood was collected 72 hours after injection and effector memory T cells ($CD44^{+}CD62L^{-}$)

in $CD8^+$ T cells were quantified by flow cytometry. *P* values: $* \leq 0.05$ by unpaired T-test. **F**-**H**, $CD8^+$ T cells sorted from naïve mice were cultured alone or with pre-plated MOC2 cells in the presence of PBS, BEMPEG, ^{90}Y-NM600, or ^{90}Y-NM600+BEMPEG together. After 30 hours incubation, (**F** and **G**) $live^+CD8^+$ T cells and (**H**) $TNF\alpha^+$ and $Ki\text{-}67^+$ in $CD8^+$ T cells were analyzed by flow cytometry. *P* values: $* \leq 0.05$; $** \leq 0.01$; $*** \leq 0.001$; $**** \leq 0.0001$; ns: not significant by one-way ANOVA with Tukey's correction for multiple comparisons (**F** and **G**) and unpaired T-test (**H**).

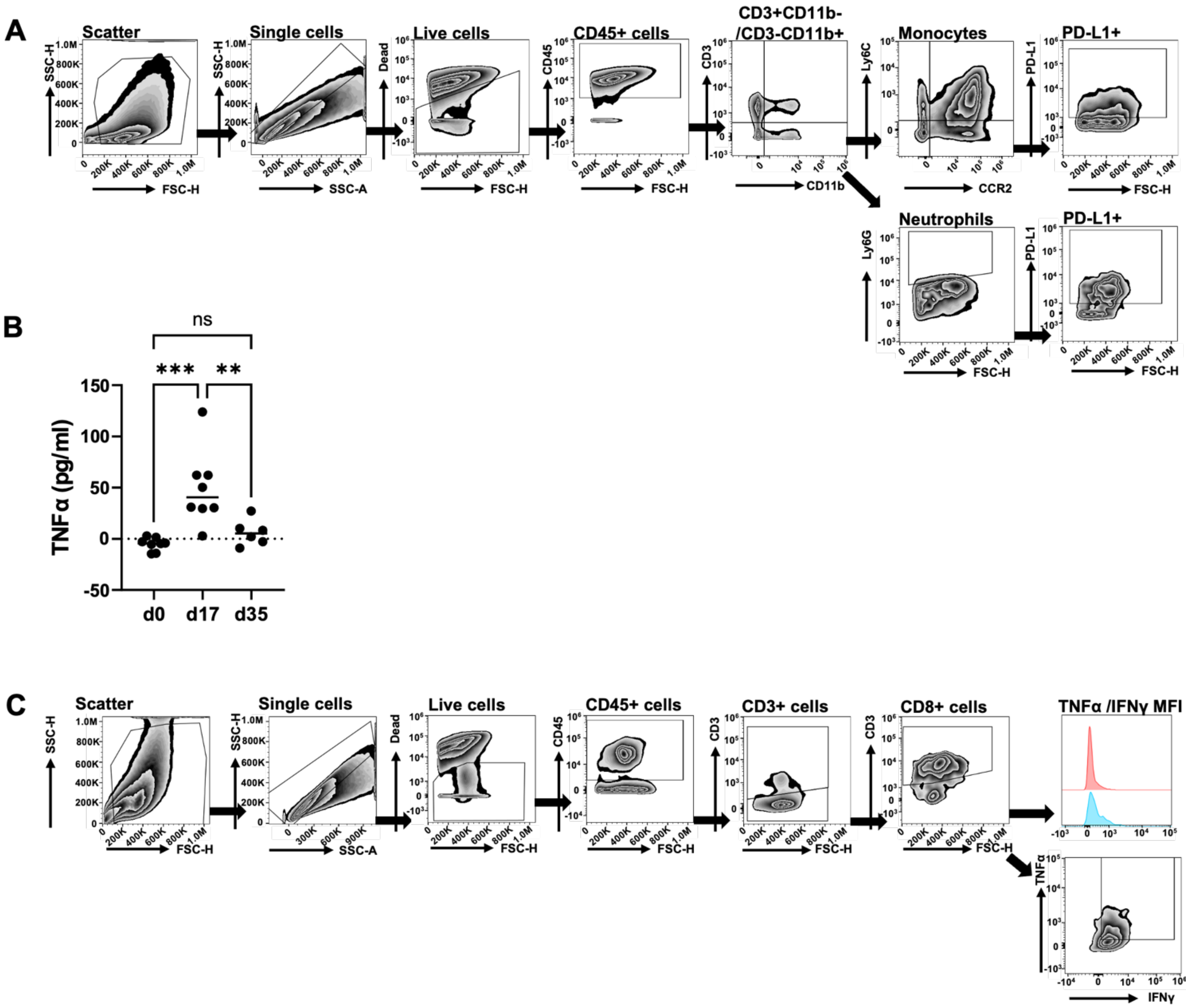

Figure S6. (**A**) Flow cytometry gating strategy. (**B**) TNFα serum concentrations from ^{90}Y-NM600 + pegIL2-treated MOC2-bearing mice at the indicated day. (n=6-8/group) *P* values: $** \le 0.01$; $*** \le 0.001$; ns: not significant by one-way ANOVA with Tukey's correction for multiple comparisons (**C**) Flow cytometry gating strategy.

Table S1. Primer sequence list

Murine Genes	Primer sequences 5' -3'	
	Forward	Reverse
CD274	CCAGCCACTTCTGAGCATGA	CTTCTCTTCCCACTCACGGG
Ifnb	CCCTATGGAGATGACGGAGA	CTGTCTGCTGGTGGAGTTCA
Oas2	TAAGAGGCTGCTCCGATGGT	GACGTCAAGGTATGCATCTTGGT
Oas3	TTTCTCAGTCAAAGGCGTCCA	TCTATCCAGTGTTCTCCGTCTG
Mx1	AGCTCACCTCCCACATCTGTAA	GCTTGCACTCTGATGACTGCTAT
Tnf	AAGTTCCCAAATGGCCTCCC	CCTAACTGCCCTTCCTCCATC
Tgfb1	CTATTGCTTCAGCTCCACAG	GACAGAAGTTGGCATGGTAG
Trex1	CCATTTCTCAGGGACTTCCA	AGCTCAGCTTTGCTCAGACC
Prf1	TTCGGGAACCAAGCTACACC	AGTAATGTGTGCAGGGGCTC
Gzmb	AAAGGCAGGGGAGATCATCG	AGGCTGCTGATCCTTGATCG
Ifng	AACAAGGCGAAAAAGGATGC	TCATTGAATGCTTGGCGCTG
Ccl2	TTTGAATGTGAAGTTGACCCGT	GTGCTTGAGGTGGTTGTGGA
Ccl3	ACATCATGAAGGTCTCCACCAC	CCATATGGCGCTGAGAAGACT
Ccl4	CCTCCCACTTCCTGCTGTTT	GTCTCATAGTAATCCATCACAAAGC
Ccl5	GAAGGAACCGCCAAGTGTGT	CCTAGCTCATCTCCAAATAGTTGAT
Cxcl10	CGCTGCAACTGCATCCATA	TAGGCTCGCAGGGATGATTTC
Cxcl11	TGTGACATCCTGGGAACGTCT	TTCCAAGACAGCAGAGGGTCA
H2-k1	GAGCTGTGGTGGCTTTTGTG	GATCAGAGGTCTGGGAGCCT
H2-d1	TGGTGCTGCAGAGCATTACA	GCGTTCCCGTTCTTCAGGTA
Hprt	AGCCTAAGATGAGCGCAAGT	GGCCACAGGACTAGAACACC

Table S2: Patient Characteristics

Characteristic	n for category (% or Range)
Sex	
Male	5 (100)
Female	0 (0)
Median age	65 y (33-79)
Histology	
SCC	5 (100)
Ethnicity	
White	5 (100)
Other	0 (0)
Smoking status	
Current/former	2 (40)
Never smoker	3 (60)
Prior therapy	
Chemoradiation	3 (60)
Surgery	2 (40)
Number of Lesions	3 (2-4)
Cycles of NKTR-214	3 (1-6)
Site of RT	
Head & Neck	2 (40)
Thorax	2 (40)
Liver	1 (20)

Abbreviation: SCC, squamous cell carcinoma

Table S3: Summary of adverse events

Adverse Events Among 5 Patients Evaluable for Toxicity					
	No. (% of patients)				
Adverse Event	Grade 1	Grade 2	Grade 3	Grade 4	Grade 5
Dry Mouth	2 (40)	0	1 (20)	0	0
Anemia	2 (40)	0	0	0	0
Constipation	1 (20)	0	0	0	0
Dry Skin	1 (20)	0	0	0	0
Eczema	1 (20)	0	0	0	0
Edema limbs	1 (20)	0	0	0	0
Edema limbs	1 (20)	0	0	0	0
Fatigue	3 (60)	0	0	0	0
Fever	1 (20)	0	0	0	0
Flu-like symptoms	1 (20)	0	0	0	0
Hyperthyroidism	2 (40)	0	1 (20)	0	0
Hypoglycemia	1 (20)	0	0	0	0
Hyponatremia	0	1 (20)	0	0	0
Hypothyroidism	0	2 (40)	0	0	0
Lipase increased	1 (20)	0	0	0	0
Lymphocyte count decreased	2 (40)	1 (20)	1 (20)	0	0
Neutrophil count decreased	1 (20)	0	0	0	0
Oral Pain	1 (20)	0	1 (20)	0	0
Pruritus	2 (40)	0	0	0	0
Rash maculo-papular	0	1 (20)	0	0	0
Thrush	0	0	1 (20)	0	0
White blood cell decreased	1 (20)	0	0	0	0
Lipase increased	1 (20)	0	0	0	0
Eosinophilia	4 (80)	0	0	0	0
Lymphocyte count decreased	0	1 (20)	0	1 (20)	0
Neutrophil count decreased	1 (20)	0	0	0	0

Table S4. EORTC QLQ-C30 Score

EORTC QLQ-30 Score (Median, %)					
Dimension	Screening	C1D8	C2D8	C3D8	30-Day Post
QL2	66.7	50	70.8	75	62.5
PF2	100	93.3	90	93.3	93.3
RF2	100	100	66.7	91.7	83.3
EF	66.7	66.7	87.5	83.3	83.3
CF	83.3	83.3	100	91.7	83.3
SF	83.3	83.3	100	91.7	91.7
FA	13.3	20	16.7	13.3	23.3
NV	0	16.7	16.7	16.7	0
PA	33.3	33.3	25	25	16.7
DY	0	0	0	0	0
SL	33.3	33.3	16.7	16.7	0
AP	33.3	33.3	16.7	16.7	33.3
CO	0	0	0	0	0
DI	0	0	0	0	16.7
FI	0	0	0	0	0

*QL2 Global health status, PF2 Physical functioning, RF2 Role functioning, EF Emotional functioning, CF Cognitive functioning, SF Social functioning, FA Fatigue, NV Nausea and vomiting, PA Pain, DY Dyspnea, SL Insomnia, AP Appetite loss, CO Constipation, DI Diarrhea, FI Financial difficulties

Table S5. EORTC QLQ-H&N35

EORTC H&N35 Scoring					
Dimension	Screening	C1D8	C2D8	C3D8	30-Day Post
HNPA	25	33.3	20.8	0	29.1
HNSW	8.3	8.3	20.8	8.3	20.8
HNSE	16.6	0	0	8.3	16.6
HNSP	11.1	0	16.6	5.5	22.2
HNSO	25	16.6	16.6	12.5	45.8
HNSC	0	0	6.6	6.6	10
HNSX	0	16.6	0	16.6	16.6
HNTE	0	0	0	0	16.6
HNOM	0	0	16.6	16.6	0
HNDR	66.6	66.6	50	50	33.3
HNSS	66.6	33.3	33.3	16.6	33.3
HNCO	33.3	66.6	33.3	16.6	50
HNFI	0	33.3	16.6	16.6	0
HNPK	60	80	75	50	50
HNNU	60	40	75	50	0
HNFE	20	20	0	0	0
HNWL	40	20	0	0	75
HNWG	40	20	25	25	0

*HNPA Pain, HNSW Swallowing, HNSE Senses problem, HNSP Speech problems, HNSO Trouble with social eating, HNSC Trouble with social contact, HNSX Less sexuality, HNTE Teeth, HNOM Opening mouth, HNDR Dry mouth, HNSS Sticky saliva, HNCO Coughing, HNFI Felt ill, HNPK pain killers, HNNU Nutritional supplements, HNFE Feeding tube, HNFL Weight loss, HNWG Weight gain

Table S6. EQ-5D Scoring

EQ-5D Score					
Dimension	Baseline n or median (%; range)	C1D8 n or median (%; range)	C2D8 n or median (%; range)	C3D8 n or median (%; range)	30 Day n or median (%; range)
Mobility					
No problems	4 (80%)	4 (80%)	4 (100%)	3 (75%)	3 (75%)
Slight problems	0	1 (20%)	0	1 (25%)	1 (25%)
Moderate problems	0	0	0	0	0
Severe problems	1 (20%)	0	0	0	0
Unable to walk about	0	0	0	0	0
Self-care					
No problems	4 (80%)	4 (80%)	4 (100%)	4 (100%)	4 (100%)
Slight problems	1 (20%)	1 (20%)	0	0	0
Moderate problems	0	0	0	0	0
Severe problems	0	0	0	0	0
Unable to wash or dress	0	0	0	0	0
Usual activities					
No problems	4 (80%)	3 (60%)	3 (75%)	3 (75%)	3 (75%)
Slight problems	0	2 (40%)	1 (25%)	1 (25%)	0
Moderate problems	0	0	0	0	1 (25%)
Severe problems	1 (20%)	0	0	0	0
Unable to do usual activities	0	0	0	0	0
Pain/discomfort					
No pain/discomfort	1 (20%)	0	2 (50%)	2 (50%)	2 (50%)
Slight pain/discomfort	2 (40%)	2 (40%)	1 (25%)	0	0
Moderate pain/discomfort	2 (40%)	3 (60%)	1 (25%)	1 (25%)	1 (25%)
Severe pain/discomfort	0	0	0	0	1 (25%)
Extreme pain/discomfort	0	0	0	1 (25%)	0
Anxiety/Depression					
Not anxious/depressed	2 (40%)	4 (60%)	2 (50%)	2 (50%)	3 (75%)
Slight anxious/depressed	3 (60%)	2 (40%)	2 (50%)	1 (25%)	1 (25%)
Moderate anxious/depressed	0	0	0	1 (25%)	0
Severe anxious/depressed	0	0	0	0	0
Extremely anxious/depressed	0	0	0	0	0
Your Health					
	81 (50-90)	65 (55-87)	87.5 (60-100)	80 (40-100)	65 (50-95)
